# Fis is a global regulator critical for modulation of virulence factor production and pathogenicity of *Dickeya zeae*

**DOI:** 10.1038/s41598-017-18578-2

**Published:** 2018-01-10

**Authors:** Mingfa Lv, Yufan Chen, Lisheng Liao, Zhibin Liang, Zurong Shi, Yingxin Tang, Sixuan Ye, Jianuan Zhou, Lianhui Zhang

**Affiliations:** 1 State Key Laboratory for Conservation and Utilization of Subtropical Agro-Bioresources, South China Agricultural University, Guangzhou, 510642 China; 2 Guangdong Province Key Laboratory of Microbial Signals and Disease Control, Integrative Microbiology Research Centre, South China Agricultural University, Guangzhou, 510642 China

## Abstract

*Dickeya zeae* is the causal agent of rice foot rot disease, which has recently become a great threat to rice planting countries and regions. The pathogen produces a family of phytotoxins named zeamines that is critical for bacterial virulence, but little is known about the signaling pathways and regulatory mechanisms that govern zeamine production. In this study, we showed that a conserved transcriptional regulator Fis is involved in the regulation of zeamine production in *D. zeae* strain EC1. Deletion mutants were markedly attenuated in the virulence against rice seed germination. Transcriptome and phenotype analyses showed that Fis is a potent global transcriptional regulator modulating various virulence traits, including production of extracellular enzymes and exopolysaccharides, swimming and swarming motility, biofilm formation and cell aggregation. DNA gel retardation analysis showed that Fis directly regulates the transcription of key virulence genes and the genes encoding Vfm quorum sensing system through DNA/protein interaction. Our findings unveil a key regulator associated with the virulence of *D. zeae* EC1, and present useful clues for further elucidation of the regulatory complex and signaling pathways which govern the virulence of this important pathogen.

## Introduction

Genus *Dickeya* bacteria massively produce extracellular degradative enzymes, such as pectate lyases, cellulases, polygalacturonases and proteases, as major virulence factors to rapidly destroy the cell tissues and invade host plants^1–4^. Among the seven species of *Dickeya*, *D*. *zeae* is unique that it can infect both dicotyledons and monocotyledons^5^, whereas the others mostly infect dicotyledons^6,7^. It is known that *D*. *zeae* strain EC1 produces a family of phytotoxins and antibiotics, named zeamines, which are capable of inhibiting rice seed germination and growth^5,8–10^. Recently, genome sequence analysis led to identification of a *zms* gene cluster encoding the biosynthesis and transportation of zeamines^11^. The *zms* cluster contains 18 ORFs, among which *zmsA* and *zmsK* have been characterized genetically and biochemically^9,10^. Mutation of *zmsA* completely abolished the zeamine production and the bacterial virulence, while the *zmsK* mutant failed to produce zeamine but produced zeamine II and maintained partial virulence against rice seed germination. These findings together indicate that zeamines are the key virulence determinants of *D*. *zeae* against rice plants. This *zms* gene cluster was only present in the *D*. *zeae* strain isolated from rice and some *D*. *solani* strains, but absent in other *Dickeya* species and the *D*. *zeae* strains isolated from other plants or sources^11^. How the *zms* genes are regulated thus becomes interesting and intriguing.

Little is known about the regulatory mechanisms that govern the virulence of *D*. *zeae*. We showed previously that the acyl-homoserine lactone (AHL) quorum sensing system plays a certain role in regulation of the virulence-related traits in *D*. *zeae* ^5^. Mutation of the *expI* gene that encodes an AHL synthase resulted in changed patterns of bacterial motility and biofilm formation, but had only a minor impact on zeamine production and bacterial virulence^5^, suggesting that the AHL quorum sensing system is not the dominant regulatory mechanism associated with the bacterial virulence. Similarly, systemic deletion analysis of the genes encode cyclic di-GMP (c-di-GMP) metabolism unveiled a few genes associated with modulation of various virulence traits including biofilm formation, bacteria motility, exoenzyme production and zeamine production in *D*. *zeae* EC1^12^, but how these c-di-GMP genes are regulated needs further investigations. A more recent study showed that the mutant of *slyA*, which encodes a SlyA/MarR family transcription regulator, is significantly decreased in zeamine production, biofilm formation and pathogenicity on rice^13^. Much work is needed to understand the regulatory mechanisms and networks associated with the virulence of this important bacterial pathogen.

In an attempt to further understand the regulatory mechanisms associated with zeamine production and transportation, we randomly picked up a range of the genes encoding transcriptional regulators based on our recently published genome sequence information of *D*. *zeae* EC1^11^, and generated corresponding mutants using gene in-frame deletion procedures. These mutants were assayed for changed patterns in zeamine production, and the promising ones were further characterized on their overall biological functions and the mechanisms of regulation. In this study, we focused on characterization of a gene encoding a transcriptional regulator belonging to the Fis family, which was named as *fis* as it is a highly conserved gene and has only one copy in the bacterial genome. Our results showed that Fis regulates zeamine production at the transcriptional level and also plays a crucial role in regulation of many other virulence related traits, such as production of extracellular degradative enzymes and exopolysaccharides, cell motility and biofilm formation. The findings from this study add a new member to the list of virulence regulators in *D*. *zeae*, and demonstrate that Fis plays a global regulatory role controlling the bacterial pathogenicity and physiology.

## Results

### Deletion of *fis* decreases the antimicrobial activity of *D*. *zeae* EC1

**Figure 1:**
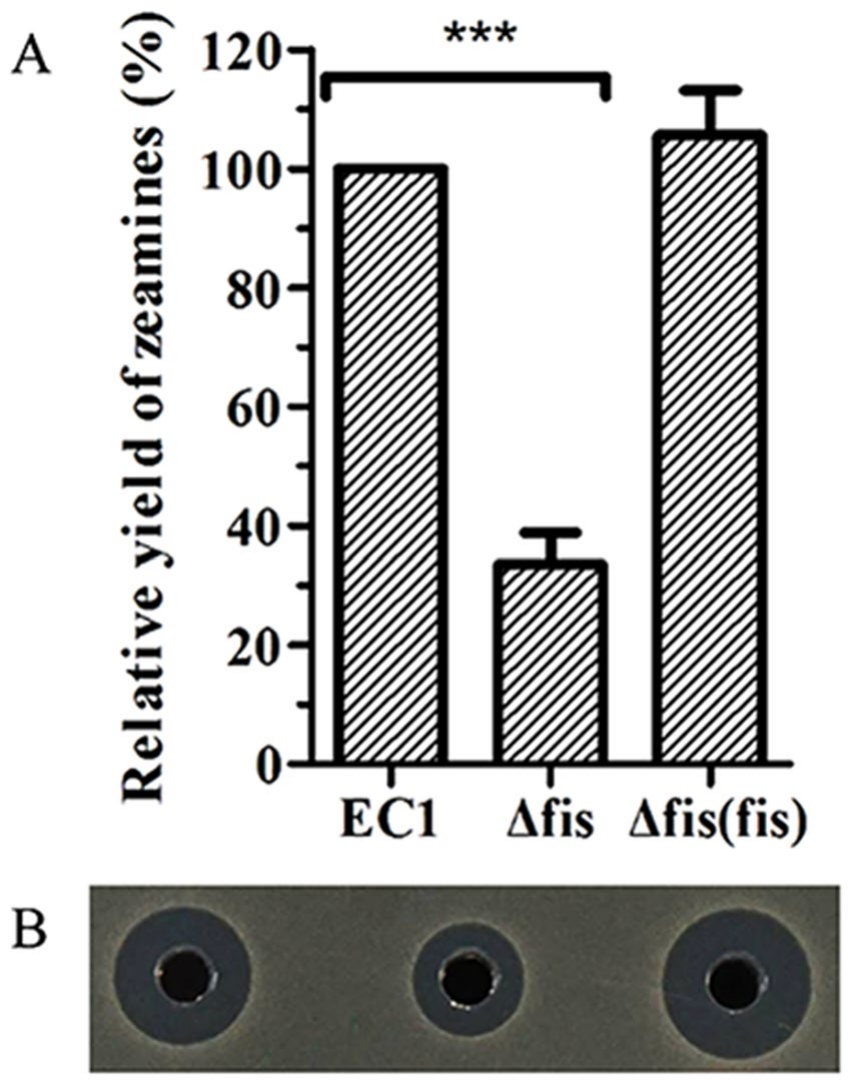
The null Fis mutation decreased the antimicrobial activity of *D*. *zeae* strain EC1. (**A**) Quantitative analysis of zeamine yield of wild-type strain EC1 and its derivative strains. The antimicrobial activity bioassay plates were prepared by pouring 15 mL of LB agar medium into the 120 × 120 mm plates, and then overlaid with 20 mL of 1% agarose containing 1.0 × 10^8^ cells of fresh *E*. *coli* harboring pBBR1MCS4 plasmid. Wells of 5 mm in diameter were punched after solidification. Overnight bacterial cultures were grown in LS5 medium to OD_600_ at around 1.4, which was centrifuged twice at 12,000 rpm for 10 min, and 20 μl of the supernatants were added into the wells. The plates were incubated at 37 °C for 10 h. The antimicrobial activity was determined by measuring the radius of the visible clear zone surrounding the well. The concentration of zeamines was determined by this formula: zeamines (unit) = 0.5484e^0.886x^ (R^2^ = 0.9957), X is the radius in millimeters of the inhibition zone surrounding the well. (**B**) Plate assay of the antimicrobial activities of wild-type strain EC1 and its derivative strains. The photograph was taken after 12 h of incubation at 37 °C. Final results of fis mutant were normalized to that of the wild-type EC1, which was set to a value of 100%, for easy comparison. Experiments were repeated at least three times in triplicates. Symbol: ***P < 0.0001 (Student’s *t*-test).

To elucidate the regulatory mechanisms involved in *D*. *zeae* virulence, we have recently completed the genome sequencing of *D*. *zeae* EC1^11^. Bioinformatics analysis showed that the genome of *D*. *zeae* EC1 encodes 185 transcriptional factors and 74 two-component system (TCS) proteins. Given the general roles of transcriptional factors and TCSs in transcriptional and translational regulation of bacterial physiology and virulence, we randomly selected over 3 dozens of the genes encoding transcriptional factors and TCSs and generated the corresponding in-frame deletion mutants, respectively, and assayed for changed patterns in zeamine production. One mutant, in which the gene (NCBI accession No.: AJC64815) encoding a conserved Fis family transcription factor was deleted from 19^th^ to 295^th^ bp of its ORF, showed over 60% reduction in zeamine production compared to its parental wild type strain EC1 (Fig. 1). *In trans* expression of the wild type *fis* gene in the deletion mutant restored zeamine production to a level similar to that by strain EC1 (Fig. 1), suggesting that it is a key regulator in modulation of zeamine production.

The ORF of *fis* consists of 297 nt encoding a peptide of 98 aa, which is highly conserved in *Dickeya* species sharing 97–100% amino acids identity with its counterparts from *D*. *paradisiaca* Ech703 (ACS87446.1), *D*. *dadantii* 3937 (NCBI accession No.: WP_012883002.1), and *D*. *solani* MK10 (WP_012883002.1). Fis is also conserved in other bacterial species such as *Escherichia coli* (NCBI accession No.: WP_044698230.1) and *Klebsiella pneumoniae* (NCBI accession No.: KTG78588.1) with 98% similarity at the amino acid level. Domain analysis showed that Fis contains a HTH_8 superfamily DNA binding domain, agreeable with its role as a nucleoid associated protein^14–16^.

### Fis is a global transcriptional regulator modulating the expression of over 13% genes in the bacterial genome

**Figure 2:**
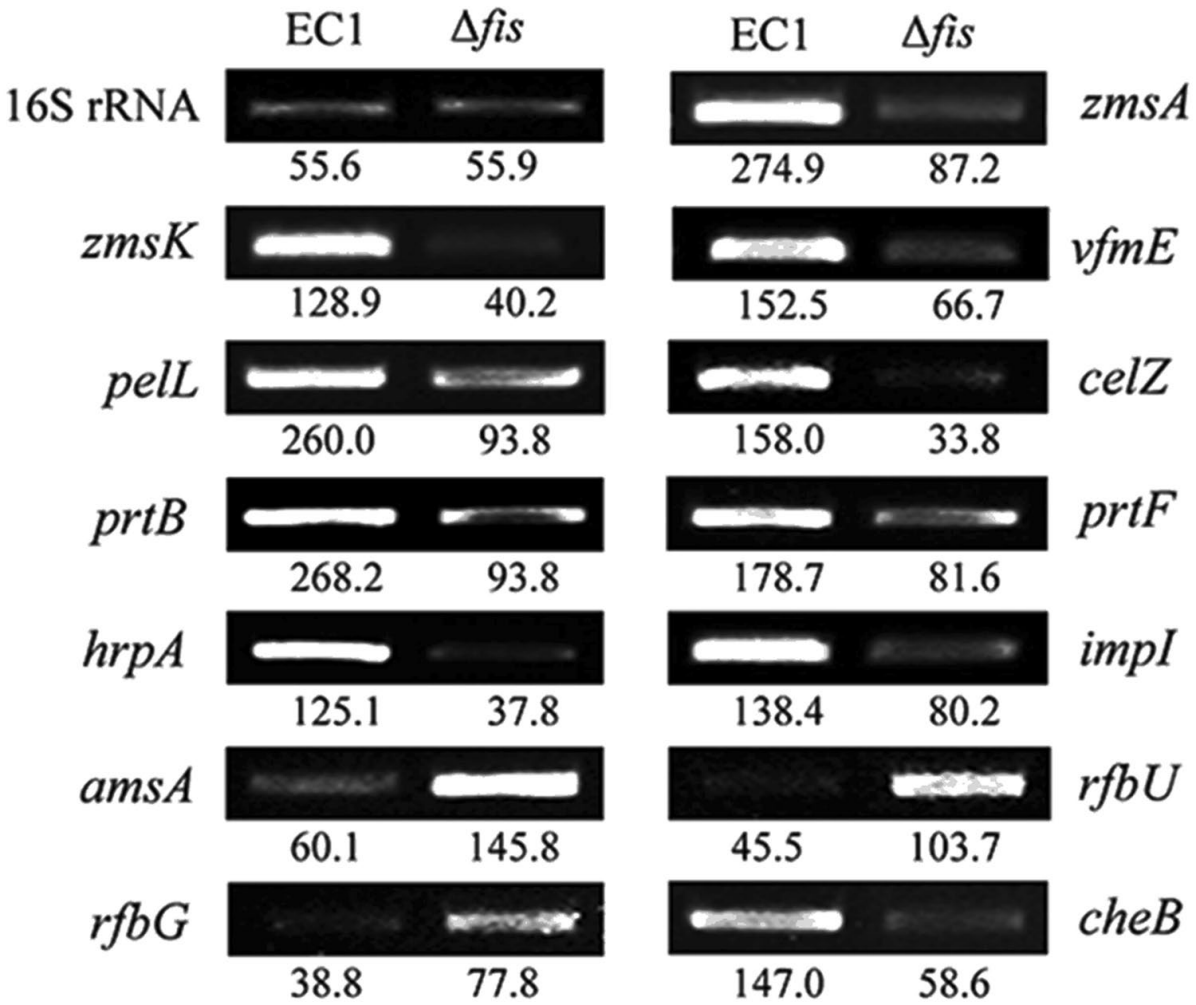
RT-PCR analysis of Fis on modulating the genes expression of major virulence factors. *zmsA* and *zmsK* encode the key virulence for biosynthesis of zeamine and zeamine II; *vfmE* encodes an AraC family transcriptional regulator; *pelL* encodes pectate lyase; *celZ* is required in the cellulase biosynthesis; *prtB* and *prtF* encode protease and a T1SS secretion protease respectively; *hrpA* encodes a type III secretion system protein (T3SS); *impI* encodes a type VI secretion system protein; *amsA*, *rfbU* and *rgpB* are involved in the biosynthesis of extracellular polysaccharides^11,20,51^. CheB is a chemotaxis protein. The reference gene of 16S rRNA was used standardizing the samples of RNA and each RNA samples, two dilutions (5 and 50 ng) were used as templates for RT-PCR reactions with a similar pattern of results. The signal intensity determined for each RNA sample using the software Image Lab (Bio-Rad, USA). Experiments were repeated three times in triplicates and the means was indicated the signal intensity for RT-PCR bands. The original photos of this figure are presented in Supplementary Fig. S1.

To further understand the role of Fis in *D*. *zeae*, we compared the transcriptome profiles of the *fis* deletion mutant and the wild type strain EC1. Of the 4,154 genes predicted in EC1 genome^11^, expression of 490 genes were significantly (|Fold Change| ≥ 2-fold, *q* ≤ 0.05) regulated by Fis, including 283 genes downregulated and 207 genes upregulated (Supplementary Table S2). These Fis-dependent genes could be grouped into 13 functional categories, including (i) zeamine synthesis, (ii) vfm (virulence factor modulated) quorum sensing, (iii) extracellular enzymes, (iv) polysaccharide synthesis, (v) secretion system, (vi) ribosomal protein, (vii) bacterial chemotaxis protein, (viii) transporters system, (ix) metabolic pathways, and (x) hypothetical proteins etc (Table 1). The reliability of the transcriptome results was verified by semiquantitative RT-PCR analysis of randomly selected genes listed in Table 1. Consistent with the transcriptome data, mutation of *fis* resulted in decreased expressions of the zeamine synthesis genes *zmsA* and *zmsK* by 3.15 and 3.21 folds, respectively. Similarly, the transcript level of the extracellular degradation enzyme genes *celZ* and *prtB* was down-regulated by 4.67 and 2.85 folds, respectively, and the type I secretion system protein *prtF* was down-regulated by 2.19-fold, whereas the transcription of *amsA*, *rfbU* and *rfbG*, which are related to EPS synthesis, were up-regulated 2.43, 2.28 and 2.01 folds, respectively (Fig. 2).

**Table 1:** Functional groups of genes differently expressed by modulation of Fis.

Gene family	Gene name or ID	Fold change (Δ*fis*/EC1)
Zeamine synthesis cluster	*zmsP*, *zmsQ*, *zmsR*, *zmsS*, *zmsA*, *zmsB*, *zmsC*, *zmsD*, *zmsE*, *zmsF*, *zmsJ*, *zmsK*, *zmsL*	−2.18 to −4.23
Vfm cluster	*vfmD*, *vfmE*, *vfmF*, *vfmG*	−2.02 to −14.69
	*vfmP*, *vfmO*, *vfmN*, *vfmM*, *vfmV*	2.38 to 5.28
Extracellular enzymes		
Cellulase	*celZ*	−2.49
Pectate lyase	*pnl*, *pelL*	−2.08 to −2.67
	*pelC*, *pelB*	3.57 to 6.97
Protease	*prtC*, *prtB*, *prtG*	−2.29 to −6.36
Proteinase inhibitor	*Inh*	−2.63
Polysaccharide synthesis	*wza*, *amsI*, *amsA*, *AJC65705*.*1*, *rfbU*, *rfbN*, *rfbB*, *rfbA*, *rfbC*, *rfbD*, *rfbG*, *AJC65715*.*1*, *manC*, *manB*	2.13 to 24.25
Secretion system		
Type I secretion	*prtE*, *prtF*	−2.12 to −4.41
Type II secretion	*outC*, *outD*, *outE*, *outF*, *outG*, *outH*, *outI*, *outJ*, *outK*	−2.03 to −4.41
Type III secretion	*AJC66388*.*1*, *hrpN*, *hrpF*, *hrpA*, *hrpJ*, *hrpQ*, *hrpO*, *hrcR*	−2.01 to −4.86
	*hrpV*, *hrcS*, *hrcT*	2.18 to 2.66
Type VI secretion	*vgrG*, *rhsA*, *rhs*, *vasD*, *impI*, *impJ*, *AJC67605*.*1*	−2.11 to −4.65
Membrane protein	*AJC65019*.*1*, *mipA*, *AJC65588*.*1*, *AJC66796*.*1*, *flk*, *AJC67186*.*1*, *AJC67231*.*1*	−2.01 to −2.60
	*AJC64703*.*1*, *AJC65094*.*1*, *elaB*, *AJC66375*.*1*, *AJC66652*.*1*, *AJC67298*.*1*, *AJC67589*.*1*	2.07 to 2.54
Ribosomal protein		
30S ribosomal protein	*rpsJ*, *rpsO*, *rpsQ*, *rpsR*, *rpsS*, *rpsU*, *rpsC*, *rpsD*	−2.09 to −3.02
50S ribosomal protein	*rplA*, *rplJ*, *rplK*, *rplM*, *rplP*, *rplS*, *rplB*, *rplV*, *rplW*, *rplY*, *rpmB*, *rpmC*, *rplC*, *rpmH*, *rpmI*, *rpmJ*, *rplD*	−2.00 to −3.88
Bacterial chemotaxis protein		
Chemotaxis	*cheB*, *cheX*, *cheY*	−2.04 to −3.18
Methyl-accepting chemotaxis protein	*AJC66063*.*1*, *AJC66982*.*1*	−2.76 to −3.09
	*AJC67591*.*1*, *AJC67164*.*1*, *AJC68205*.*1*	2.23 to 2.87
Transporters		
Iron complex transport system	*fepC*, *fbpB*, *fepD*, *yvrC*, *fbpA*, *fepB*, *feoA*, *FeoB*, *AJC67280*.*1*, *nark*, *metI*	−2.11 to −6.12
ABC transporter binding protein	*ssuB*, *oppD*, *AJC67079*.*1*, *AJC67279*.*1*, *AJC67282*.*1*	−2.20 to −3.37
	*AJC64678*.*1*, *bztD*, *rbsB*, *AJC67793*.*1*, *bztA*, *araF*, *gsiA*, *AJC67001*.*1*, *mglA*	2.04 to 4.08
ABC transporter permease	*livH*, *AJC67570*.*1*, *potC*	2.08 to 3.74
Branched-chain amino acid transport system	*AJC67074*.*1*	2.25
Oligogalacturonide transport system	*togA*, *togM*, *togN*	−2.20 to −2.56
	*AJC66045*.*1*, *ugpE*	2.07 to 2.89
Other transporter	*lysE*, *AJC65268*.*1*, *cbrB*, *exbD*, *exbB*, *AJC64909*.*1*, *AJC65810*.*1*, *AJC65018*.*1*	−2.00 to −3.18
	*AJC64601*.*1*, *AJC65873*.*1*, *phnE*, *malK*, *hycB*	2.08 to 2.62
Metabolic pathways	*rpoC*, *birA*, *purT*, *purE*, *paaG*, *fabG*, *csdA*, *ectB*, *pldA*	−2.00 to −3.52
	*araA*, *AJC67397*.*1*, *araB*, *araD*, *pyrE*, *aceB*, *AJC65547*.*1*, *yhdh*, *iolE*, *AJC65065*.*1*	2.11 to 3.99
Phosphotransferase system (PTS)	*celB*, *celC*, *celA*	−2.08 to −3.46
	*manX*, *manY*, *manZ*, *ulaB*	2.07 to 2.54
Nitrate reductase and transporter	*narI*, *narJ*, *narH*, *narZ*, *nark*, *narX*, *narL*	−2.50 to −9.85
Transcriptional regulator	*AJC64976*.*1*, *acrR*, *AJC67225*.*1*, *AJC67942*.*1*	−2.50 to −6.43
	*AJC66654*.*1*, *AJC68091*.*1*	2.02 to 3.02
Indigoidine biosynthetic Genes	*indC*	−2.05
Hypothetical protein	*AJC68312*.*1*, *AJC68320*.*1*, *AJC64956*.*1*, *AJC68327*.*1*, *AJC68351*.*1*, *AJC65351*.*1*, *AJC65686*.*1*, *AJC65733*.*1*, *AJC65740*.*1*, *AJC65741*.*1*, *AJC65742*.*1*, *AJC68373*.*1*, *AJC65745*.*1*, *AJC65747*.*1*, *AJC65777*.*1*, *AJC68376*.*1*, *AJC68377*.*1*, *AJC68380*.*1*, *AJC65843*.*1*, *AJC68389*.*1*, *AJC68391*.*1*, *AJC65937*.*1*, *AJC65961*.*1*, *AJC65962*.*1*, *AJC66009*.*1*, *AJC68394*.*1*, *AJC68397*.*1*, *AJC66128*.*1*, *AJC66158*.*1*, *AJC66175*.*1*, *AJC66182*.*1*, *AJC66398*.*1*, *AJC66462*.*1*, *AJC66464*.*1*, *AJC66471*.*1*, *AJC66490*.*1*, *AJC66501*.*1*, *AJC66508*.*1*, *AJC68422*.*1*, *AJC66551*.*1*, *AJC66579*.*1*, *AJC66708*.*1*, *AJC66777*.*1*, *AJC66880*.*1*, *AJC66881*.*1*, *AJC66882*.*1*, *AJC66885*.*1*, *AJC66887*.*1*, *AJC66917*.*1*, *AJC66922*.*1*, *AJC66924*.*1*, *AJC66925*.*1*, *AJC66926*.*1*, *AJC68442*.*1*, *AJC66960*.*1*, *AJC68452*.*1*, *AJC67230*.*1*, *AJC67234*.*1*, *AJC67511*.*1*, *AJC68470*.*1*, *AJC67598*.*1*, *AJC67599*.*1*, *AJC67609*.*1*, *AJC68474*.*1*, *AJC67696*.*1*, *AJC67697*.*1*, *AJC67698*.*1*, *AJC68479*.*1*, *AJC67813*.*1*, *AJC67816*.*1*, *AJC67943*.*1*	−2.00 to −23.48
	*AJC64584*.*1*, *AJC64664*.*1*, *AJC64666*.*1*, *AJC64701*.*1*, *AJC68315*.*1*, *AJC64784*.*1*, *AJC64806*.*1*, *AJC68323*.*1*, *AJC65050*.*1*, *AJC65063*.*1*, *AJC65435*.*1*, *AJC65471*.*1*, *AJC65609*.*1*, *AJC65723*.*1*, *AJC65724*.*1*, *AJC65727*.*1*, *AJC68374*.*1*, *AJC66033*.*1*, *AJC66034*.*1*, *AJC68406*.*1*, *AJC68411*.*1*, *AJC66216*.*1*, *AJC66223*.*1*, *AJC66420*.*1*, *AJC66583*.*1*, *AJC68427*.*1*, *AJC66653*.*1*, *AJC66700*.*1*, *AJC68434*.*1*, *AJC66755*.*1*, *AJC67176*.*1*, *AJC67182*.*1*, *AJC67519*.*1*	2.01 to 21.82

### Deletion of *fis* resulted in reduced extracellular degradative enzymes

**Figure 3:**
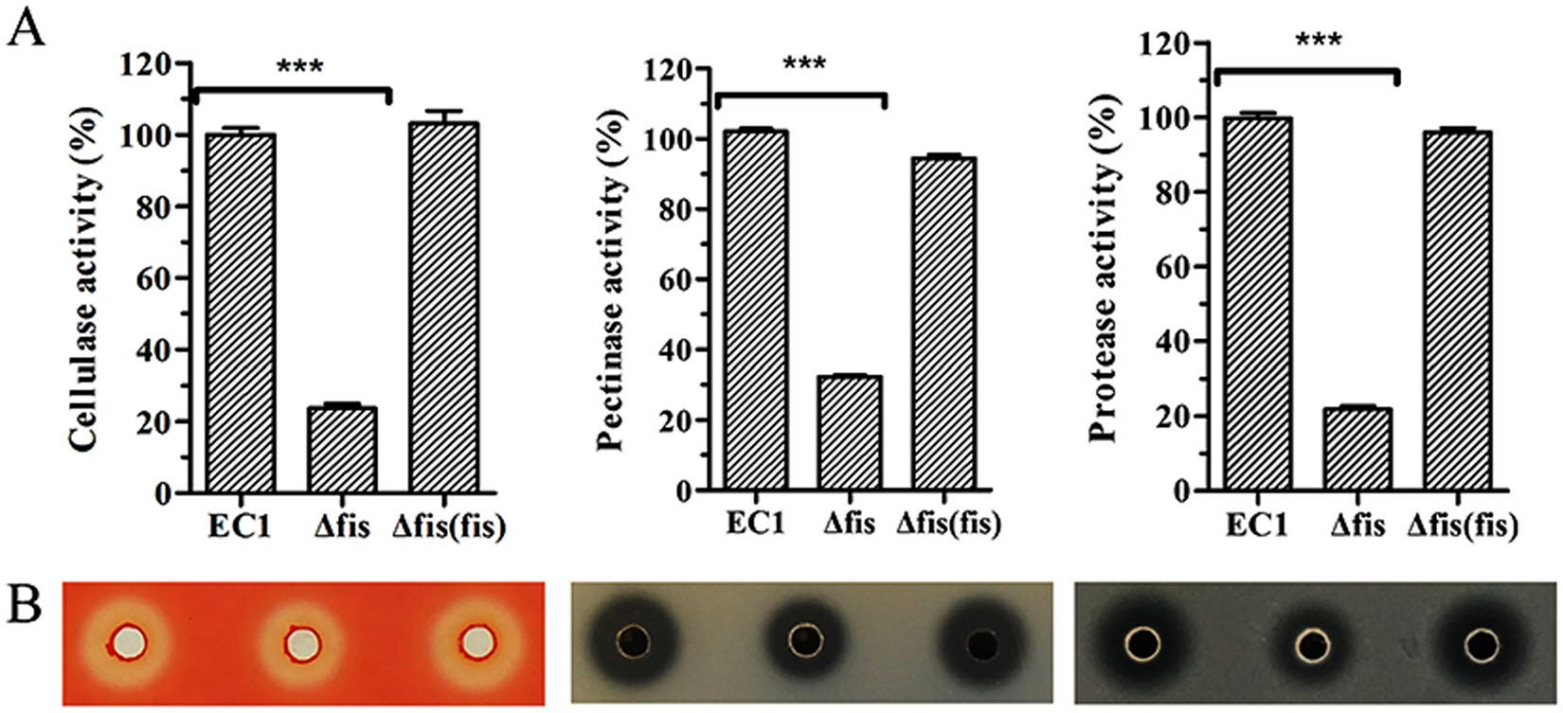
Deletion of *fis* resulted in decreased production of extracellular enzymes. (**A**) Quantitative measurement of extracellular enzymes activity for wild-type strain EC1 and derivatives. The quantitative determination, the protease activity was determined through measuring the absorbance at 440 nm with azocasein as substrate; the cellulose activity was determined through absorbed spectrum value under 550 nm with carboxymethylcellulose sodium as substrate and 3,5-dinitrosalicylic acid as colorant; the pectate lyase activity was determined via the absorbed spectrum value under 235 nm with polygalacturonic acid as substrate. The experiment was repeated three times. The data were the means of three repeats and the standard deviation is represented using error bar. (**B**) Extracellular enzymes production on bioassay plates. Twenty microlitres of culture supernatants of wild-type strain EC1 and its derivatives strains were added into the wells on assay plates and incubated at 28 °C. Final results of *fis* mutant were normalized to that of the wild-type EC1, which was set to a value of 100%, for easy comparison. Experiments were repeated at least three times in triplicates. Symbol: ***P < 0.0001 (Student’s *t*-test).

Extracellular degradative enzymes (pectinases, polygalacturonases, cellulases and proteases) were secreted by *Dickeya* spp. to degrade the structure of host plant cells and result in the soft rot symptoms^1,17,18^. Following the clues from the transcriptome analysis, we tested the extracellular enzyme activities of *fis* mutant and its derivatives. Quantitative enzyme activity analysis showed that the mutant had a lower ability to digest the substrates of pectate lyases, cellulases and proteases than wild-type strain EC1 with cellulase, pectate lyases and protease activities decreased about 78%, 68%, and 78%, respectively, which were restored to the wild type levels by *in trans* expression of the *fis* gene in the deletion mutant (Fig. 3A). The above results were confirmed by enzyme plate assays, which showed that Fis positively regulated the production of these three groups of exoenzymes (Fig. 3B). Similarly, RT-PCR analysis results showed that the expression of the genes *celZ*, *prtB* and *prtF*, which are involved in the synthesis or secretion of cellulase and protease, respectively, in the *fis* deletion mutant were substantially decreased (Fig. 2).

### Deletion of *fis* enhanced the production of extracellular polysaccharides

**Figure 4:**
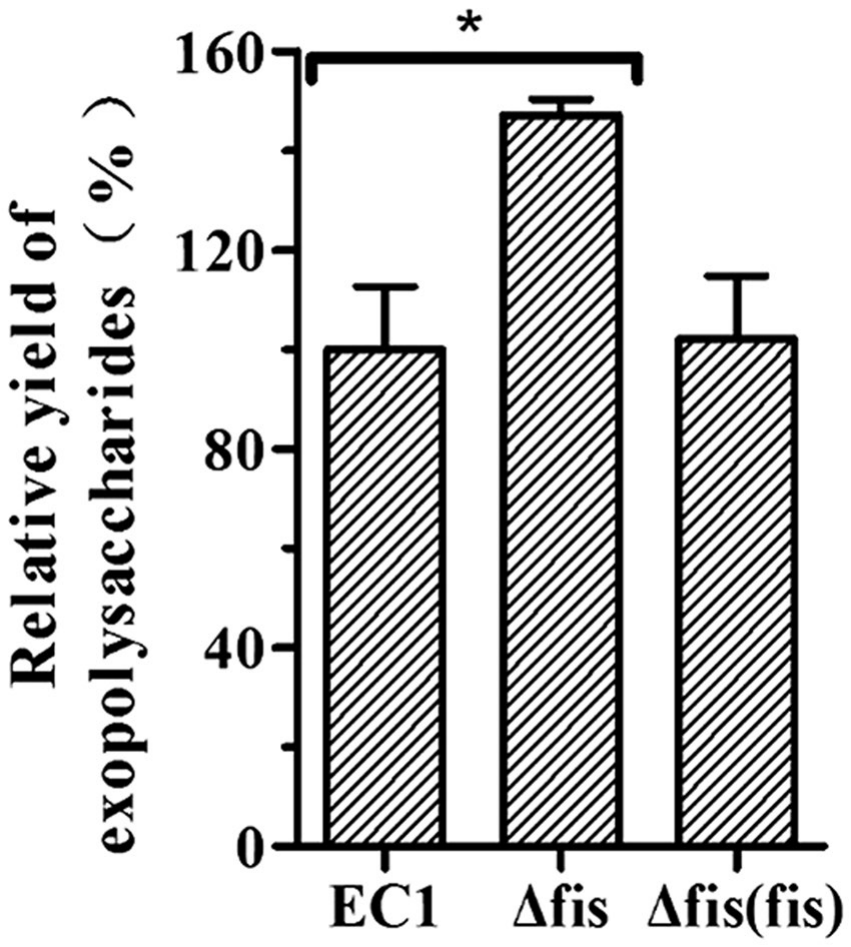
Quantitative analysis of EPS production in *D*. *zeae* wild-type strain EC1 and derivatives. Ten mililiters of overnight bacterial cultures in LB medium at a density of OD_600_ = 1.8 were centrifuged at 12,000 rpm for 15 min. Two volumes (20 ml) of absolute ethanol were added into the collected supernatants and thoroughly mixed before incubation for 1 h at 4 °C. EPS was isolated by centrifuging at 12,000 rpm for 30 min at 4 °C and dried overnight at 55 °C before determination of the dry weights. Final results of *fis* mutant were normalized to that of the wild-type EC1, which was set to a value of 100%, for easy comparison. Experiments were repeated at least three times in triplicates. *P < 0.05 (Student’s *t*-test).

Extracellular polysaccharide (EPS) is one of the major virulence factors for many phytopathogenic bacteria^19^. The transcriptome data indicate that the expression of the genes associated with EPS biosynthesis, including the genes *ams*, *wza*, *rfb*, and *manB*/*C* ^20–23^, were increased markedly upon deletion of the *fis* gene (Table 1). To determine the impact of Fis on the production of EPS, we determined the EPS yields of the *fis* mutant and the wild-type strain EC1. Results showed that EPS product was significantly increased in *fis* mutant, and fully recovered to the EC1 level in the complemented strain (Fig. 4). The transcriptome and enzyme assay data are highly consistent with the RT-PCR results (Fig. 2). Therefore, it is established that Fis negatively regulates the EPS biosynthesis in *D*. *zeae* EC1.

### Mutation of *fis* reduced the bacterial motility

**Figure 5:**
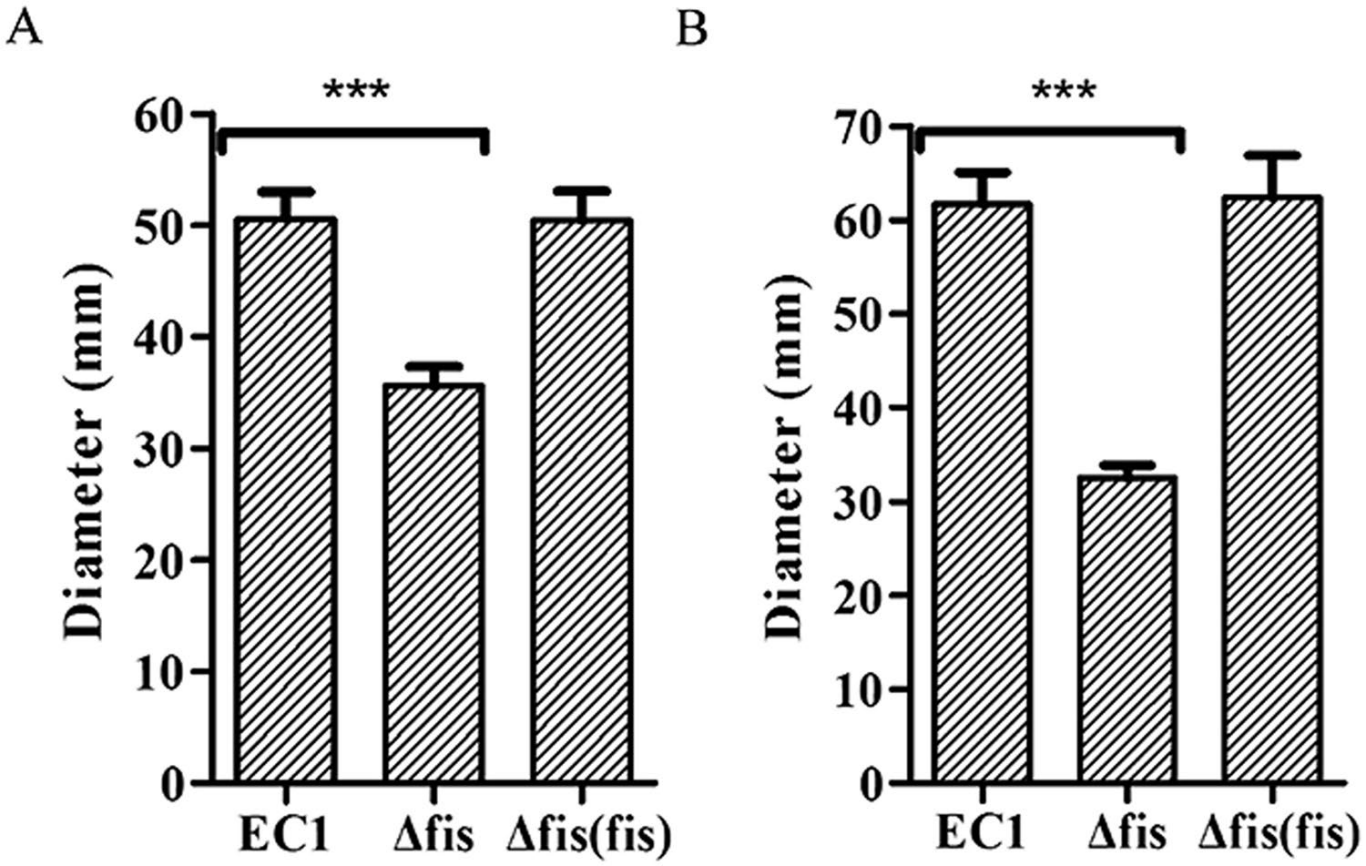
Mutation of *fis* gene reduced cell motility. The photographs of swimming and swarming plates were taken 40 h and 14 h after inoculation. (**A**) Swimming motility of strain EC1 and its derivatives. (**B**) Swarming motility of strain EC1 and its derivatives. Experiments were repeated at least three times in triplicates. Symbol: ***P < 0.0001 (Student’s *t*-test).

Swimming and swarming are two different types of motility in bacteria require the presence of bacterial flagella and influenced by other factors such as EPS and chemotaxis. This study showed that the swimming motility of the *fis* mutant was reduced by about 30% in comparison with wild-type EC1 and the complemented strain (Fig. 5A). Similarly, the swarming motility of the *fis* mutant was about 47% less than that of the wild-type EC1, while the complemented strain had the same level of swarming motility as strain EC1 (Fig. 5B).

The transcriptome analysis showed no significant difference in the expression of bacterial flagella encoding genes in the *fis* mutant and the wild type strain EC1. However, the expressions of genes encoding chemotaxis signal transduction protein CheB, CheX and CheY were reduced by 2.04, 3.18 and 2.29 folds, respectively, in the *fis* deletion mutant compared to strain EC1 (Table 1), which were reported to be associated with the motility in at least some bacterial species^24–26^. The RT-PCR results support the data of transcriptome analysis (Fig. 2), and seem to be agreeable with the results of bacterial motility assay. In addition, increased EPS production is also known to accompany decreased bacterial motility^27^.

### Biofilm formation is decreased and cell aggregation increased in the *fis* mutant

**Figure 6:**
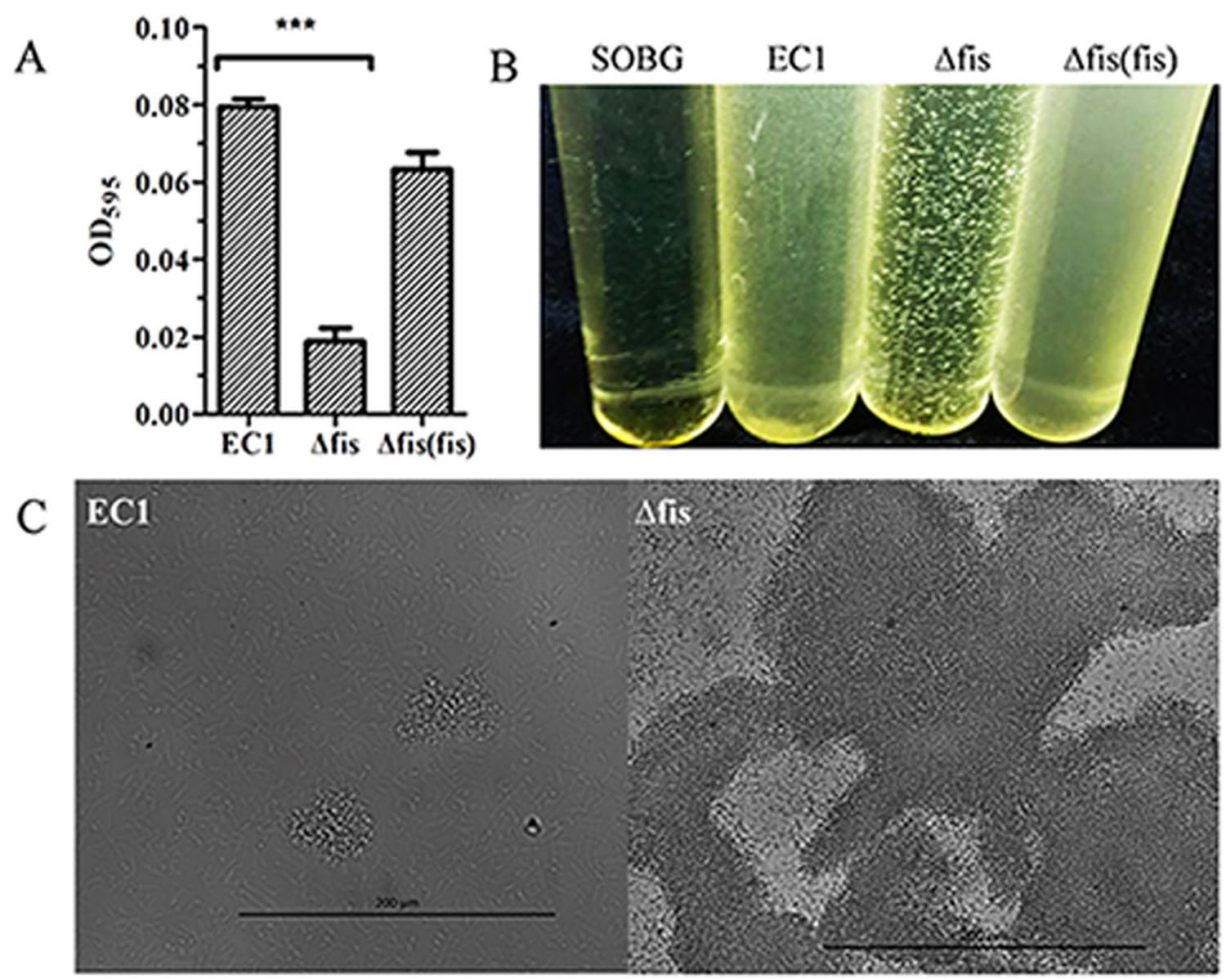
Deletion of *fis* decreased the biofilm formation and enhanced cell aggregation. (**A**) Quantitative characterization of biofilm formation of *D*. *zeae* wild-type strain EC1 and derivatives. Experiments were repeated at least three times in triplicates. (**B**) Cell aggregation of *D*. *zeae* wild-type strain EC1 and derivatives. Bacteria were grown at 28 °C with 200 rpm shaking in SOBG medium for 6 h. Photograph was taken after growth for 6 h. (**C**) Microscopy inspection of cell aggregates of strain EC1 and the *fis* mutant, respectively. Photographs were taken at x400 under a Nicon H550S microscope. Symbol: ***P < 0.0001 (Student’s *t*-test).

Biofilm is important for bacteria to survive in harsh environment. To investigate the function of Fis in modulating biofilm (attached bacterial cells) formation, we used 96-well plates for quantitative measurement. Data showed that the *fis* deletion mutant had lower ability to form surface-attached biofilms in SOBG (SOB plus glycerol) medium than the wild type strain EC1, whereas the complemented strain partially recovered the biofilm formation (Fig. 6A).

Interestingly, we found that obvious clumps of bacterial cells were observed in the culture of *fis* mutant, but not in strain EC1 and the complemented strain (Fig. 6B). We hence conducted a microscopic analysis using wild type strain EC1 and the *fis* mutant. The results showed that only a few small cell clumps of strain EC1 observed under microscope, whereas the *fis* mutant formed much bigger cell clumps under the same condition (Fig. 6C).

### Deletion of *fis* decreased the bacterial virulence on rice seed germination

**Figure 7:**
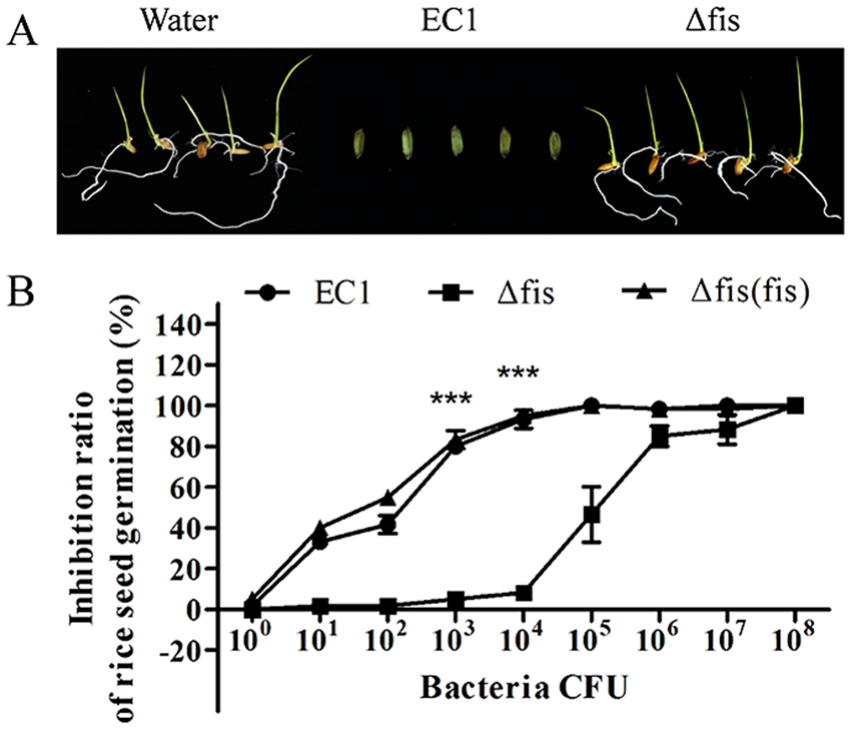
Deletion of *fis* in strain EC1 abolished its inhibitory activity on rice seed germination. Water was used as a control. (**A**) Photographs of rice seed germination treated with strain EC1 and its derivatives at a final concentration of inocula at 10^4^ CFU. The photographs were taken 7 days after inoculation. (**B**) Quantitative analysis of the inhibitory activity of strain EC1 and its derivatives on rice seed germination. Rice seeds were treated with different bacterial dilutions as indicated, and incubated at 28 °C for 7 days with light of 16 h and darkness of 8 h. Experiments were repeated at least three times in triplicates. The paired two-tailed Student’s *t*-test was performed between the wild type EC1 and *fis* mutant under the 10^3^ and 10^4^ bacteria CFU respectively. Symbol: ***P < 0.0001.

Previous study indicate that zeamines are one of the major virulence factors in strain EC1^9,10^. Transcriptome and RT-PCR analysis showed that the expression of *zmsA* and *zmsK* was reduced significantly in the *fis* mutant compared to strain EC1 (Table 1; Fig. 2), suggesting that Fis might exert substantial influence on the bacterial virulence. Consistent with these findings, virulence assay results showed that deletion of *fis* resulted in loss of bacterial virulence against rice seed germination when rice seeds were incubated with bacterial solution at 10^3^ CFU (Fig. 7A). Further quantitative assay with different concentrations of bacterial inocula showed that inhibition rate of rice seed germination was about 93% by strain EC1 and 95% by the complemented strain Δ*fis*(*fis*) when challenged with the pathogen at 10^4^ CFU. In contrast, the inhibition rate of the mutant Δ*fis* on rice seed germination was just only 8% under the same conditions. When the inoculum concentration was increased to 10^5^ CFU, strain EC1 and the complemented strain Δ*fis*(*fis*) completely inhibited the rice seed germination, but the mutant Δ*fis* had showed only about 50% of inhibition. The *fis* mutant reached a complete inhibition of rice seed germination at 10^8^ CFU (Fig. 7B), suggesting that null mutation of Fis led to over 1000-fold reduction in bacterial virulence against rice seed germination.

### Fis regulates the transcription of virulence genes through protein-promoter interaction

**Figure 8:**
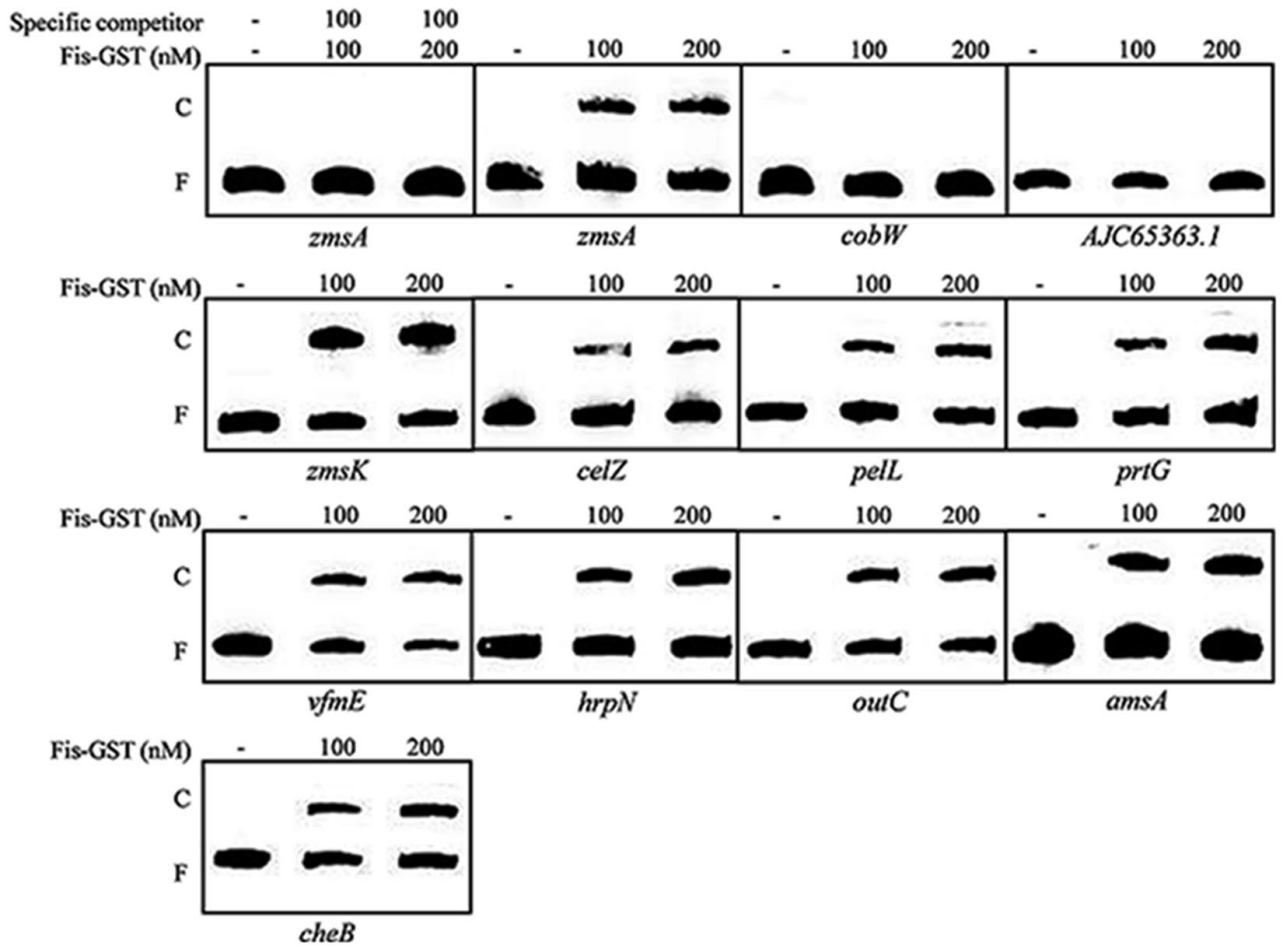
EMSA analysis of Fis protein and promoter DNA interactions. Thirty fmol of labelled DNA sequences corresponding to the promoter region of *zmsA*, *zmsK*, *celZ*, *pelL*, *prtG*, *vfmE*, *hrpN*, *outC*, *amsA* and *cheB* genes were incubation with 100 nM and 200 nM Fis-GST, respectively, using 100-fold unlabeled corresponding DNA fragments as the specific competitor. The promoters of *cobW* and *AJC65363*.*1*, which are not regulated by Fis according to transcriptome data, were used as negative controls in the EMSA experiment. The positions of free DNA (F) and of protein-DNA complexes (C) are shown. The original photographs of this figure are presented in Supplementary Fig. S3.

Given that Fis is a DNA binding protein, we selected a range of virulence related genes based on transcriptome analysis data, and tested whether Fis could bind to their promoters through electrophoretic motility shift assay (EMSA). The selected genes include *zmsA* and *zmsK* involved in synthesis of zeamines; *vfmE* encodes an AraC family transcriptional regulator associated with the biosynthesis of a novel QS signal^28^; the *prtGDEFBCX* cluster encodes four proteases and three protease secretion associated proteins; *celZ* encodes an endoglucanase and is involved in cellulose degradation; *pelL* product is involved in pectin degradation and encode pectate lyases; *hrpN* encode a T3SS effector; *cheB*, *cheX* and *cheY* encode proteins associated with chemotaxis and bacterial motility; the *outSBCDEFGHIJKLMO* encodes the Out system (Type II secretion system, T2SS); and *amsA* is involved in the biosynthesis of extracellular polysaccharides^11,29–32^. The promoter regions of these genes were amplified by PCR, respectively, and the Fis protein was purified through affinity chromatography by generating an in-frame fusion with the coding region of glutathione S-transferase (GST). The results demonstrate the specific binding of Fis to the promoter regions of *zmsA*, *zmsK*, *vfmE*, *celZ*, *pelL*, *prtG*, *cheB*, *hrpN*, *amsA* and *outC* (Fig. 8), whereas GST failed to bind to these promoters (Supplementary Fig. S2). As a negative control, Fis could not bind to the promoter of *cobW* and *AJC65363*.*1*, which encodes cobalamin biosynthesis protein and hypothetical protein and are not regulated by Fis according to our transcriptome analysis results (Fig. 8).

## Discussion

Fis protein was originally identified as a factor for inversion stimulation of the homologous Hin and Gin site-specific DNA recombinases of *Salmonella* and phage Mu^33^. It is an important small nucleotide-associated protein which plays a role in affecting the bacterial chromosome structure and the initiation of DNA replication^34,35^. In the closely related bacterial species *D*. *dadantii*, Fis was shown to modulate the production of the extracellular enzymes, including celllulases, pectate lyases, and proteases, and biofilm formation^36–38^. Similarly, our results in this study showed that Fis is also involved in regulation of extracellular enzyme production and biofilm formation in *D*. *zeae* EC1 (Figs 2, 3, 6A and 8), suggesting a high level of conservation in the biological functions modulated by this global regulator. Importantly, this study unveils several new functions regulated by Fis, including zeamine production, EPS biosynthesis, and expression of Type III and Type VI secretion systems (Table 1; Figs 1, 2, 4 and 8) Of the 18 ORFs in the *zms* gene cluster encoding zeamine biosynthesis and related functions^11^, the transcripts of 14 genes were significantly reduced in the *fis* mutant (Tables 1 and S2). Consistent with the results of genetic analysis, phenotype assay showed that deletion of *fis* significantly decreased zeamine production and reduced the inhibitory activity against rice seed germination by three orders of magnitude compared to the wild-type strain EC1 (Fig. 7). Moreover, transcriptome analysis showed that Fis regulated the transcriptional expression of 567 genes belonging to 13 functional groups (Table 1). The reliability of the transcriptome data was further validated by RT-PCR and gel retardation analyses (Figs 2 and 8). Taken together, these findings suggest that *D*. *zeae* heavily relies on Fis in modulation of the bacterial physiology and pathogenicity.

Similar to the AHL quorum sensing system and the transcriptional regulator SlyA, Fis is also involved in the regulation of bacterial motility and biofilm formation. However, they appear to act in different ways. While mutation of the AHL synthase gene *expI* and *slyA* resulted in increased swimming and swarming motility^5,13^, deletion of *fis* led to decreased swimming and swarming motility (Fig. 5). This is contrary to their regulatory mode on zeamine biosynthesis, in which deletion of both *slyA* and *fis* caused a significant reduction in zeamine production^13^. It is highly intriguing how these two transcriptional regulators could inversely regulate the bacterial motility and reciprocally control the zeamine biosynthesis, which is worthy of further investigations. Transcriptome analysis unveils that Fis could modulate the transcriptional expression of a range of regulators, including 6 transcriptional regulators (*AJC64976*.*1*, *acrR*, *AJC67225*.*1*, *AJC67942*.*1*, *AJC66654*.*1* and *AJC68091*.*1*) and 9 genes encoding the Vfm quorum sensing system (*vfmD*, *vfmE*, *vfmF*, *vfmG*, *vfmP*, *vfmO*, *vfmN*, *vfmM* and *vfmV*) (Table 1). Vfm was firstly identified in *D*. *dadantii* 3937 as a new quorum sensing system to modulate the production of extracellular enzymes^28^. Genome sequence analysis showed that the *vfm* gene cluster is also present and conserved in *D*. *zeae* EC1^11^. The results from this study showed that among the 9 *vfm* genes influenced by Fis, 4 are positively regulated and 5 are negatively regulated by Fis (Table 1), suggesting that the *vfm* gene cluster may contain several transcriptional units. Significantly, expression of *vfmE*, which encodes a transcriptional regulator, was decreased by about 14-fold, and that of *vfmF* and *vfmG* was decreased by about 2-fold in the *fis* mutant compared to its wild type *D*. *zeae* EC1 (Table 1). It is believed that *vfmE* expression is activated by the signal receptor kinase VfmI and its cognate response regulator VfmH upon detecting extracellular Vfm signals, and VfmE then activates both the transcription of cell wall-degrading enzymes and the *vfm* operon^28^. The results of this study showed that Fis protein can bind to the promoter of *vfmE* and control its transcription (Table 1) (Fig. 8). This suggests that the Vfm quorum sensing system may be located at the downstream of Fis regulator, which is contrast to the case of DSF quorum sensing system in *Xanthomonas campestris*, where the global regulator Clp is acting in the middle of DSF signaling pathway^39^.

In summary, the findings from this study demonstrate that the global regulator Fis plays a key role in regulation of the virulence and physiology in *D*. *zeae* EC1. In particular, we showed that Fis positively regulates the biosynthesis of zeamine, which is the key virulence factor of *D*. *zeae* EC1, by direct interaction with the promoter of *zmsA*. Identification of Fis and the recently reported SlyA^13^ as the key regulators of zeamine biosynthesis provides useful toolkits and platforms for further understanding the regulatory networks and signaling mechanisms that govern the virulence of *D*. *zeae*. In addition, given that zeamines are potent antibiotics against both bacterial and fungal pathogens^8,40,41^, the above findings also present useful clues for synthetic engineering *D*. *zeae* strains to increase the yield of zeamines.

## Materials and Methods

### Bacterial strains and growth conditions

The strains used in this study were listed in Table 2. *D*. *zeae* strain EC1 and its derivatives were cultivated at 28 °C in minimal medium broth (MM)^10^, LS5 medium^40^ and Luria-Bertani (LB) medium as indicated. *E*. *coli* strains were grown at 37 °C in LB medium. Antibiotics were added to the medium at the following final concentrations when required: polymyxin B sulfate, 25 µg/ml; ampicillin, 100 µg/ml; streptomycin, 50 µg/ml; and kanamycin, 50 µg/ml.

**Table 2:** Strains and plasmids used in this study.

Strains or plasmids	Relevant phenotypes and characteristics^a^	Source or reference
Strains		
EC1	Wild type of *Dickeya zeae*, PB^r^	Lab collection
Δ*fis*	A deletion mutant derived from EC1	This research
Δ*fis*(*fis*)	Δfis containing *fis* coding region at the downstream of *lacZ* promoter, Amp^r^, PB^r^	This research
CC118λ	*Escherichia coli* strain as host for plasmid constructs derived from pKNG101	Lab collection
DH5α	*E*. *coli* strain as host for plasmid constructs derived from pBBR1-MCS4	Lab collection
HB101(pRK2013)	*Thr leu thi recA hsdR hsdM pro*, Kan^r^	Lab collection
Plasmids		
pKNG101	Knockout vector, Str^r^	Lab collection
pKNG101-*fis*	pKNG101 carries the in-frame deleted fragment of *fis*, Str^r^	This research
pGEX-6p-*fis*	pGEX-6p-1 carries the *fis* coding region, Amp^r^	This research
pBBR1-MCS4	Expression vector contains a *lacZ* promoter, Amp^r^	This research
pBBR1-*fis*	pBBR1-MCS4 carries the coding region of *fis* at down-stream of *lacZ* promoter, Amp^r^	This research

^a^PB^r^, Amp^r^, Kan^r^, Str^r^ = resistance to Polymyxin B Sulfate, Ampicillin, Kanamycin, or Streptomycin, respectively.

### Gene deletion and complementation

Plasmid pKNG101 listed in Table 2 was used in this study to generate the deletion mutants of *D*. *zeae* EC1. To generate the deletion mutant of *fis* in the genetic background of *D*. *zeae* strain EC1, the 5′- and 3′- flanking regions of the *fis* gene were respectively amplified using two primer pairs, i.e., *fis*-1/*fis*-2, and *fis*-3/*fis*-4, with the genomic DNA of strain EC1 as template. Subsequently, the two flanking fragments of the *fis* gene were fused by overlapping extension PCR using primers *fis*-1 and *fis*-4. The PCR products were purified with AxyPrep^TM^ DNA gel extraction kit (AXYGEN) and digested with *Bam*HI and *Spe*I restriction endonucleases and then cloned into the suicide plasmid pKNG101 digested with the same restriction endonucleases^42,43^. The resultant gene deletion construct was transformed into *E*. *coli* CC118λ competent cells by heat shock at 42 °C and introduced into strain EC1 through triparental mating. In triparental mating, the donor and receptor cells were mixed with the helper strain *E*. *coli* RK2013 in a ratio of 2:1:1 on LB plate and incubated at 28 °C for 8 h. The transformants were spread and grown in MM medium containing streptomycin and polymyxin B sulfate. The colonies were then spread on MM agar plates containing 5% sucrose and polymyxin B sulfate to exclude the suicide plasmid. The resultant *fis* deletion mutants were confirmed by PCR using the detection primer pair *fis*-F and *fis*-R.

For complementation, the coding region of *fis* was amplified from the EC1 genomic DNA using the primer pair C*fis*-H/C*fis*-B. The PCR products were purified and digested with *Hind*III and *Bam*HI restriction endonucleases and then cloned at the downstream of the *lac* promoter in the vector pBBRI-MCS4 digested with corresponding restriction endonucleases. The complementary construct was transformed into *E*. *coli* DH5α and detected using the primers MCS-F/MCS-R designed based on the flanking sequences of the cloning sites of pBBRI-MCS4 and determined by DNA sequencing. The confirmed construct was introduced into the *fis* deletion mutant by triparental mating as described above and confirmed by PCR using the primers MCS-F/MCS-R.

### Extracellular enzyme activity assays

The cellulase, pectate lyase and proteolytic enzyme activities were determined using carboxymethyl cellulose sodium, polygalacturonic acid and skimmed milk as substrates, respectively, following the methods described previously^44,45^. The extracellular enzymes activities were measured as follows: assay plates were prepared by pouring about 35 ml of substrate medium into the 120 × 120 mm petri dishes, and wells of 5 mm in diameter were punched in the assay plates after solidification; bacteria were cultured overnight at 28 °C in LB medium when the bacterial population density was reached about OD_600_ = 1.4; the supernatants of 20 μl were taken and added into the wells of the assay plates^44^, and incubated at 28 °C and then plates were stained with dye as indicated. The cellulase (Cel) assay plates incubated at 28 °C for 14 h were stained with 0.1% Congo red for 10 min and then decolored with 1M NaCl for 15 min twice. The pectate lyase (Pel) plate was treated with 1 M HCl for coloration after 11 h incubation under the same temperature. The transparent zone surrounding the wells of the protease (Prt) assay plates were recorded after incubation for 24 h. For quantitative determination, the protease activity was determined through the absorbance at 440 nm with azocasein as substrate; the cellulose activity was determined by measuring at 550 nm with carboxymethylcellulose sodium as substrate and 3,5-dinitrosalicylic acid as colorant; the pectate lyase activity was determined via the absorbance at 235 nm with polygalacturonic acid as substrate. The experiment was repeated three times with triplicates each time.

### Biofilm formation and cell aggregation

Biofilm formation was measured as described previously^46–48^. Bacterial cultures were grown overnight in LB medium and diluted in SOBG medium to a density at OD_600_ = 0.01. An aliquot of 100 μl bacterial dilutions was added into each well of 96-well microtitre plate and incubated at 28 °C with shaking at 150 rpm for 18 h. The liquid cultures were eliminated and 150 μl of 1% crystal violet (w/v) were added to each well. After staining at room temperature for 20 min, the dye solutions were removed and the wells were washed three times with water. After drying under open air, the wells were added with 200 μl of 95% ethanol to dissolve the dye for quantification of the attached bacterial cells (biofilms). Quantification of the crystal violet was performed by measuring the spectrophotometric values at 595 nm with a microplate reader (BioTek).

Cell aggregation was determined by growing the overnight cultures of wild-type strain EC1 and its derivatives at 28 °C with 200 rpm shaking in SOBG medium, diluting them to 0.05 of OD_600_ and shaking for 6 h under the same condition as the above. The photos were taken at x400 under a Nicon H550S microscope and the growth statues *in vitro* was visualized by a Sony camera.

### Extracellular polysaccharide (EPS) assay

Ten mililiters of overnight bacterial cultures in LB medium at a density of OD_600_ = 1.8 were centrifuged at 12,000 rpm for 15 min. Two volumes (20 ml) of absolute ethanol were added into the collected supernatants and thoroughly mixed before incubation for 1 h at 4 °C. EPS was isolated by centrifuging at 12,000 rpm for 30 min at 4 °C and dried overnight at 55 °C before determination of the dry weights.

### Measurement of antimicrobial activity and quantification of zeamines

The antimicrobial activity bioassay plates were prepared by pouring 15 mL of LB agar medium into the 120 × 120 mm plates, and then overlaid with 20 mL of 1% agarose containing 1.0 × 10^8^ cells of fresh *E*. *coli* harboring pBBR1MCS4 plasmid. Wells of 5 mm in diameter were punched after solidification. Overnight bacterial cultures were grown in LS5 medium^40^ to OD_600_ at around 1.4, and 20 μl of the supernatants were added into the wells. The plates were incubated at 37 °C for 10 h. The antimicrobial activity was determined by measuring the radius of the visible clear zone surrounding the well. The concentration of zeamines was determined by this formula: zeamines (unit) = 0.5484e^0.886x^, the correlation coefficient is 0.9957 and X is the radius in millimeters of the inhibition zone surrounding the well^9,10,40^.

### Swimming and swarming motility

For determination of swimming motility, the plates were prepared by pouring 20 ml of semisolid Bacto tryptone agar medium (per liter contains Bacto-peptone 5 g, NaCl 5 g, and agar 3 g) into the 90 mm petri dish, and spotted with bacteria using a toothpick and inoculated at 28 °C for 40 h. The diameter of bacterial zone was measured. The swarming motility was assayed in the same condition except the medium (per liter Tryptone 5 g, NaCl 5 g, and agar 4 g) and different inoculation time at 14 h.

### RNA purification and transcriptome analysis

Overnight cultures of strain EC1 and its derivatives were diluted in fresh LB medium and cultivated at 28 °C to reach OD_600_ = 1.0. The RNA samples were prepared using the SV total RNA isolated system kit (Promega), and RNA samples were further purified using the RNA clean kit (Qiagen).

The quantity of RNA measured using a NanoDrop, Wilmington, DE ND-100 spectrophotometer and the integrality of RNA measured using an agarose gel electrophoresis. The total RNA samples were treated with DNase I to degrade any possible DNA contamination. Ribosomal RNA (rRNA) was removed using Ribo-Zero^TM^ rRNA Removal Kit (Illumina, USA) following the manufacturer’s protocol and mRNA was cleaned and enriched using the RNeasy MinElute cleanup kit (Qiagen). The mRNA samples were fragmented into short fragments after mixing with fragmentation buffer, and cDNA fragments were synthesized using random hexamer-primers, dNTPs, RNase H, and DNA polymerase I. The ends of the purified double-strand cDNA were repaired by adding adenine before purification of cDNA fragments with a QIAQuick Kit (QIAGEN, Germany). Sequencing adaptors were ligated to the fragments and PCR amplification was then performed to enrich the fragments. Finally, a library of cDNAs was constructed and sequenced on an Illumina Hisq2500 platform (CA) using PE100 strategy. Sequenced Reads were obtained by base calling using CASAVA software and saved in FASTQ format. Reads were mapped to a merged whole genome sequence from *D*. *zeae* EC1 (CP006929.1). All the raw sequencing reads have been submitted to the NCBI Sequence Read Archive, which are available under the accession number SRR5834263. The gene expression level was calculated by using RPKM (Reads per Kilobase of Transcript per Million Reads Mapped) method^49^ and analyzed using DEseq^50^. The genes with statistically significant changes in expression (|log_2_Ratio| ≥ 1 and *q* ≤ 0.05) were selected.

Reverse transcription-polymerase chain reaction (RT-PCR) was performed by StarScript II first-strand cDNA synthesis Mix (GenStar). Primer pairs were listed in the Supplementary Table S1. For each RNA samples, two dilutions (5 and 50 ng) were used as templates for RT-PCR reactions with a similar pattern of results. The signal intensity of each RT-PCR band was determined using the software Image Lab (Bio-Rad, USA).

### Rice seed germination assay

For rice seed germination assay, the overnight cultures of bacterial strains were grown in LB medium until the optical density OD_600_ reached about 1.2 and then diluted to a concentration of 1 × 10^4^ (CFU)/ml with sterile water unless otherwise indicated at 27 °C. Twenty rice seeds were put into the tube containing 5 ml of diluted cultures and incubated for 5 h in room temperature. The rice seeds were rinsed three times with sterile water and transferred onto three pieces of filter paper on a sterilized dish, and 5 ml of sterile water were added into the dish to keep moisture. The germination rate was determined after incubation at 28 °C with 16 h of light and 8 h of darkness for 7 days. Rice seeds treated with sterile water were used as the negative control. The experiment was repeated for at least twice and each time with two duplicates.

### Electrophoretic mobility shift assay (EMSA)

The coding region of *fis* was amplified by PCR using primers pGEX-6p-*fis*-F and pGEX-6p-*fis*-R containing *BamH*I and *Sal*I restriction enzyme sites, respectively (Table S1). The resultant 297 bp fragments of PCR product were cloned into the *BamH*I-*Sal*I region of vector pGEX-6p-1 by the ClonExpress^@^MultiS (Vazyme) to generate pGEX-6p-*fis* (Table 2) and fused to a Gst•tag on its N-terminus. The Fis-GST protein was over-produced in *E*. *coli* BL21 by shaking at 250 rpm at 37 °C in LB medium containing ampicillin till OD_600_ = 0.8. The cells were transferred to 18 °C, shaking at 250 rpm for overnight with IPTG added at a final concentration of 1 mM to induce Fis expression. When the bacterial cell density reaching OD_600_ at 0.8, cells were collected by centrifugation at 5,000 rpm for 10 min and resuspended in an appropriate volume of extraction buffer (Clontech). The bacteria cells were disrupted by using the Stansted Fluid Power at 120 psi and the crude extracts were prepared by centrifugation at 13,000 rpm for 20 min. Protein purification was performed at 4 °C according to the Glutathione Resin User Manual (Clontech). The protein samples were packaged in 1.5 ml centrifuge tube and stored at −80 °C till further use.

The promoter regions of target genes were amplified in a size of 300–400 bp by PCR using the primers listed in Table S1. The purified PCR fragments were labeled by biotin using the Biotin 3′ End DNA Labeling Kit (Thermo). Binding reactions were performed in a final volume of 20 μl using LightShift^@^ C*hemiluminescent* EMSA Kit following the manufacturer’s protocol (Thermo). For each reaction, 10× Binding Buffer 2 μl, 1 μg/μl Poly (dI•dC) 1 μl, Fis proteins at 100 nM or 200 nM were placed in ice-bath for 5 min before added into 30 fmol labelled DNA fragments, and incubated for 30 min at 22 °C. The protein-DNA complexes and the unbound free DNA fragments were separated on 6% nondenaturing polyacrylamide [acrylamide/bisacrylamide 29: 1 (v/v)] gels using the electrophoresis buffer TBE, and were detected by using chemiluminescence (Tanon). A 100-fold molar excess of unlabeled DNA fragments were incubated with Fis proteins for 15 min before addition of the labeled DNA fragments to verify specific interaction of the Fis protein-DNA fragments.

### Statistic analysis

Experiments were conducted with triplicates and repeated for at least twice. For easy comparison, some data of the *fis* mutant were normalized to those of the wild-type EC1, which were set to a value of 100%. The paired two-tailed Student’s t-test was performed between the wild type EC1 and its derivatives by using the GraphPad Prism 5.0 software (GraphPad, La Jolla, CA). Symbol: *P < 0.05; ***P < 0.0001.

## Electronic supplementary material

Supplementary Information

## References

[1] Hugouvieux-Cotte-Pattat N, Condemine G, Nasser W, Reverchon S (1996). Regulation of pectinolysis in *Erwinia chrysanthemi*. Annu Rev Microbiol.

[2] Sepulchre JA, Reverchon S, Nasser W (2007). Modeling the onset of virulence in a *Pectinolytic bacterium*. J Theor Biol.

[3] Kepseu WD, Sepulchre JA, Reverchon S, Nasser W (2010). Toward a quantitative modeling of the synthesis of the pectate lyases, essential virulence factors in *Dickeya dadantii*. J Biol Chem.

[4] Reverchon S, Van Gijsegem F, Effantin G, Zghidi-Abouzid O, Nasser W (2010). Systematic targeted mutagenesis of the MarR/SlyA family members of *Dickeya dadantii* 3937 reveals a role for MfbR in the modulation of virulence gene expression in response to acidic pH. Mol Microbiol.

[5] Hussain MBBM (2008). The acyl-homoserine lactone-type quorum-sensing system modulates cell motility and virulence of *Erwinia chrysanthemi* Pv. *zeae*. J Bacteriol.

[6] Sinha SK, Prasad M (1977). Bacterial stalk rot of maize, its symptoms and host-range. Zentralbl Bakteriol Parasitenkd Infektionskr Hyg.

[7] Nassar A, Bertheau Y, Dervin C, Narcy JP, Lemattre M (1994). Ribotyping of *Erwinia chrysanthemi* strains in relation to their pathogenic and geographic distribution. Appl Environ Microbiol.

[8] Wu J (2010). 13^C^ labeling reveals multiple amination reactions in the biosynthesis of a novel polyketide polyamine antibiotic zeamine from *Dickeya zeae*. Chem Commun (Camb).

[9] Zhou J (2011). A novel multidomain polyketide synthase is essential for zeamine production and the virulence of *Dickeya zeae*. Mol Plant Microbe Interact.

[10] Cheng Y (2013). A nonribosomal peptide synthase containing a stand-alone condensation domain is essential for phytotoxin zeamine biosynthesis. Mol Plant Microbe Interact.

[11] Zhou J (2015). The complete genome sequence of *Dickeya zeae* EC1 reveals substantial divergence from other *Dickeya* strains and species. BMC Genomics.

[12] Chen Y (2016). Genetic modulation of c-di-GMP turnover affects multiple virulence traits and bacterial virulence in rice pathogen *Dickeya zeae*. PLoS One.

[13] Zhou J (2016). SlyA regulates phytotoxin production and virulence in *Dickeya zeae* EC1. Mol Plant Pathol.

[14] Pan CQ (1996). Variable structures of Fis-DNA complexes determined by flanking DNA-protein contacts. J Mol Biol.

[15] Feldman-Cohen LS (2006). Common and variable contributions of Fis residues to high-affinity binding at different DNA sequences. J Bacteriol.

[16] McLean, M. M., Chang, Y., Dhar, G., Heiss, J. K. & Johnson, R. C. Multiple interfaces between a serine recombinase and an enhancer control site-specific DNA inversion. *Elife* **2**, (2013). 10.7554/eLife.01211 PMC3798978 24151546

[17] Grenier AM (2006). The phytopathogen *Dickeya dadantii* (*Erwinia chrysanthemi* 3937) is a pathogen of the pea aphid. Appl Environ Microbiol.

[18] Ma B (2007). Host range and molecular phylogenies of the soft rot enterobacterial genera *Pectobacterium* and *Dickeya*. Phytopathology.

[19] Bernhard F, Coplin DL, Geider K (1993). A gene cluster for amylovoran synthesis in *Erwinia amylovora*: characterization and relationship to *cps* genes in *Erwinia stewartii*. Mol Gen Genet.

[20] Smits TH (2013). Phylogenetic position and virulence apparatus of the pear flower necrosis pathogen *Erwinia piriflorinigrans* CFBP 5888T as assessed by comparative genomics. Syst Appl Microbiol.

[21] Case ED (2014). The Francisella O-antigen mediates survival in the macrophage cytosol via autophagy avoidance. Cell Microbiol.

[22] Dunstan RA (2015). Assembly of the secretion pores GspD, Wza and CsgG into bacterial outer membranes does not require the Omp85 proteins BamA or TamA. Mol Microbiol.

[23] Zivkovic M (2015). Exopolysaccharide production and ropy phenotype are determined by two gene clusters in putative probiotic strain lactobacillus paraplantarum BGCG11. Appl Environ Microbiol.

[24] Parales RE, Harwood CS (2002). Bacterial chemotaxis to pollutants and plant-derived aromatic molecules. Curr Opin Microbiol.

[25] Alexandre G, Zhulin IB (2003). Different evolutionary constraints on chemotaxis proteins CheW and CheY revealed by heterologous expression studies and protein sequence analysis. J Bacteriol.

[26] Parkinson JS (2003). Bacterial chemotaxis: A new player in response regulator dephosphorylation. J Bacteriol.

[27] Liu A (2016). Exopolysaccharides play a role in the swarming of the benthic bacterium *Pseudoalteromonas* sp. SM9913. Front Microbiol.

[28] Nasser W (2013). Vfm a new quorum sensing system controls the virulence of *Dickeya dadantii*. Environ Microbiol.

[29] Park SY (2004). Structure and function of an unusual family of protein phosphatases: the bacterial chemotaxis proteins CheC and CheX. Mol Cell.

[30] Yap MN, Yang CH, Barak JD, Jahn CE, Charkowski AO (2005). The *Erwinia chrysanthemi* type III secretion system is required for multicellular behavior. J Bacteriol.

[31] Muff TJ, Ordal GW (2007). Assays for CheC, FliY, and CheX as representatives of response regulator phosphatases. Methods Enzymol.

[32] Piqué N, Minana-Galbis D, Merino S, Tomas JM (2015). Virulence factors of *Erwinia amylovora*: A review. Int J Mol Sci.

[33] Koch C, Kahmann R (1986). Purification and properties of the *Escherichia coli* host factor required for inversion of the G segment in bacteriophage Mu. J Biol Chem.

[34] Wold S, Crooke E, Skarstad K (1996). The *Escherichia coli* Fis protein prevents initiation of DNA replication from *oriC in vitro*. Nucleic Acids Res.

[35] Chintakayala K (2013). *E*. *Coli* Fis protein insulates the *cbpA* gene from uncontrolled transcription. PLoS Genet.

[36] Lautier T, Nasser W (2007). The DNA nucleoid-associated protein Fis co-ordinates the expression of the main virulence genes in the phytopathogenic bacterium *Erwinia chrysanthemi*. Mol Microbiol.

[37] Ouafa ZA, Reverchon S, Lautier T, Muskhelishvili G, Nasser W (2012). The nucleoid-associated proteins H-NS and FIS modulate the DNA supercoiling response of the Pel genes, the major virulence factors in the plant pathogen bacterium *Dickeya dadantii*. Nucleic Acids Res.

[38] Prigent-Combaret C (2012). The nucleoid-associated protein Fis directly modulates the synthesis of cellulose, an essential component of pellicle-biofilms in the phytopathogenic bacterium *Dickeya dadantii*. Mol Microbiol.

[39] He Y (2007). *Xanthomonas campestris* cell-cell communication involves a putative nucleotide receptor protein Clp and a hierarchical signalling network. Mol Microbiol.

[40] Liao L (2014). Production of novel antibiotics zeamines through optimizing *Dickeya zeae* fermentation conditions. PLoS One.

[41] Liao L (2015). Control of litchi downy blight by zeamines produced by *Dickeya zeae*. Sci Rep.

[42] Kaniga K, Delor I, Cornelis GR (1991). A wide-host-range suicide vector for improving reverse genetics in gram-negative bacteria: inactivation of the *blaA* gene of *Yersinia enterocolitica*. Gene.

[43] Hoang TT, Karkhoff-Schweizer RR, Kutchma AJ, Schweizer HP (1998). A broad-host-range Flp-FRT recombination system for site-specific excision of chromosomally-located DNA sequences: application for isolation of unmarked *Pseudomonas aeruginosa* mutants. Gene.

[44] Chatterjee A, Cui Y, Liu Y, Dumenyo CK, Chatterjee AK (1995). Inactivation of *rsmA* leads to overproduction of extracellular pectinases, cellulases, and proteases in *Erwinia carotovora* subsp. *carotovora* in the absence of the starvation/cell density-sensing signal, N-(3-oxohexanoyl)-L-homoserine lactone. Appl Environ Microbiol.

[45] Caldas C, Cherqui A, Pereira A, Simoes N (2002). Purification and characterization of an extracellular protease from *Xenorhabdus nematophila* involved in insect immunosuppression. Appl Environ Microbiol.

[46] Burova LA, Ravdonikas LE, Christensen P, Schalen C, Totolian AA (1983). The genetic control of virulence in group a streptococci. II. Trigger effect by plasmids on anti-phagocytic activity, opacity factor and IgG and IgA Fc-receptors. Acta Pathol Microbiol Immunol Scand B.

[47] Dong YH, Zhang XF, An SW, Xu JL, Zhang LH (2008). A novel two-component system BqsS-BqsR modulates quorum sensing-dependent biofilm decay in *Pseudomonas aeruginosa*. Commun Integr Biol.

[48] Deng Y (2014). Diffusible signal factor (DSF) quorum sensing signal and structurally related molecules enhance the antimicrobial efficacy of antibiotics against some bacterial pathogens. BMC Microbiol.

[49] Wagner GP, Kin K, Lynch VJ (2012). Measurement of mRNA abundance using RNA-seq data: RPKM measure is inconsistent among samples. Theory Biosci.

[50] Wang L, Feng Z, Wang X, Zhang X (2010). DEGseq: an R package for identifying differentially expressed genes from RNA-seq data. Bioinformatics..

[51] Monreal D (2003). Characterization of *Brucella abortus* O-polysaccharide and core lipopolysaccharide mutants and demonstration that a complete core is required for rough vaccines to be efficient against *Brucella abortus* and *Brucella ovis* in the mouse model. Infect Immun.

