# Supplementary material for: Fis is a global regulator critical for modulation of virulence factor production and pathogenicity of Dickeya zeae
Source: Sci Rep. 2018 Jan 10;8:341. doi: 10.1038/s41598-017-18578-2 (PMC5762655; doi:10.1038/s41598-017-18578-2)
Supplement: Supplementary file 1 — Supplementary Information [file 41598_2017_18578_MOESM1_ESM.pdf]

Supplementary Information

Fis is a global regulator critical for modulation of virulence factor production and pathogenicity of *Dickeya zeae*

Ming-fa Lv[1,2], Yufan Chen[1,2], Lisheng Liao[2], Zhibing Liang[1,2], Zurong Shi[1,2], Yingxin Tang[2], Sixuan Ye[2], Jianuan Zhou[2], Lianhui Zhang[1,2]*

[1]State Key Laboratory for Conservation and Utilization of Subtropical Agro-Bioresources, [2]Guangdong Province Key Laboratory of Microbial Signals and Disease Control, Integrative Microbiology Research Centre, South China Agricultural University, Guangzhou 510642, China.

Content:

Table S1. Primers used in this study

Table S2. List of the genes regulated by Fis

Figure S1

Figure S2

Figure S3

Table S1. Primers used in this study

Primers	Sequence (5'-3')
Mutants construction primers	
fis-F	AGAATCAACGCCGAAAGCGGTG
fis-R	AAACCGCACTAAGAGCGCGAGA
fis-1	CGGGATCCCAGTATCCAGCGTTGGTCAAGC
fis-2	CAGAGATGAACGGATGCGTACACGCGTTGTTCGAACATAG
fis-3	CTATGTTCGAACAACGCGTGTACGCATCCGTTCATCTCTG
fis-4	GGACTAGTATAACGCTGGTATCTCCCCCCT
pGEX-6p-*fis*-F	ttccaggggcccctgggatccATGTTCGAACAACGCGTGAAT
pGEX-6p-*fis*-R	gatgcggccgctcgagtcgacTCAGTTCATGCCGTATTTTTTCA
C*fis*-H	CCCAAGCTTATCCAGCGTTGGTC
C*fis*-B	CGGGATCCAGGAGGCTCGTGTCTGT
MCS-F	GGCTCGTATGTTGTGTGG
MCS-R	AGCTGGCGTAATAGCGAAGA
pKNG-F	GACACTGAATACGGGGCAAC
pKNG-R	CCCCTGGATTTCACTGATGA
RT-PCR primers	
16s-F	GGCCTAACACATGCAAGTCG
16s-R	GAGTTAGCCGGTGCTTCTTC
zmsA-F	GTCGGAGGTCGATTTTTACC
zmsA-R	ATCTGATCTGGCGTAGAAGG
zmsK-F	GATCAATGGGCGTTACAAGA
zmsK-R	GGCTTGCAGTACATTCTCTC
vfmE-F	TCAGGACATGTATCGTCGTC
vfmE-R	CGAAAATGATGTCGAAGCTC
celZ-F	AACGAACCACTCCAGGTATC
celZ-R	CAGGGTAATAGGTGGAAGCC
amsA-F	TTACTGGAAAAACGCGTCAC
amsA-R	CCGATCTTTCTTCTGTTGCC
rfbU-F	GATTGTGGGTAAGAAAGGCG
rfbU-R	ACGTAACGTTTCAGATGCAC
rfbG-F	CATGTGTGTGATGACGGTTC
rfbG-R	GCATCGGTTCTATCAACTGC
impI-F	CTGGATATCAACTCGCAGGT
impI-R	ACGGGTCATTAAATCGGTCC
hrpA-F	CATAATGCGTTTATCAGA
hrpA-R	CTGTATTTCAACCCTTAC
cheB-F	AAAGACTTGGTAAATCCT
cheB-R	TTATGTGTTGATGATTCTG
prtB-F	CTATTCCACGGATAAAGCGGTG
prtB-R	GGTATATCGCCCGTATTCTTCG
prtF-F	CAATAGGGTTATCTCTGACGGG
PrtF-R	GATAGAGTCGGACCATCAAGTC
PelL-F	TTAACTCAGACGCTTACCGC

Primers	Sequence (5'-3')
PelL-R	ATTGCCAAATGCAACAGAGC
DNA sequences amplification of promoters	
zmsA-f	ATTCAGTGCTGCTGTGGTTTC
zmsA-r	TCCGACAATTGCAATGTCACTTG
zmsK-f	GCAGGATGCTTTTAAGACGG
zmsK-r	TGAATATCCAGCTCAATTAATTCA
vfmE-f	TTTCCATCCCGCTTTATCCC
vfmE-r	TCATGGTGTCATCCCAAAAAAATAG
celZ-f	TCACGCTAATCTCTACAGGTG
celZ-r	GAATGAATCTCCATTTCAGATGG
prtG-f	TGTGTTATCAGACTATGCTGCC
prtG-r	CATCACGCTTCCTCCATGAAATTAC
pelL-f	GAGTGAGGAATGGCTAAAAATG
pelL-r	CATTTATGTTGTAAAAATCATTTATCC
amsA-f	GGGAAACCGGCCGATGAA
amsA-r	GGCAATTCTCTCTGACATCGTAATCC
outC-f	CTGGCATAACAGGAAACAGGA
outC-r	CATCTGCAAACATTATCCCTATG
hrpA-f	TGTGCTAACGATTAACAGCGA
hrpA-r	GCCGATATGCTGGCTATCAG
hrpN-f	GTATGACGGATGCCACCTG
hrpN-r	CATAATCTCGTTTCCTCATTCATG
cheB-f	CGGGTATTTATCGGCAGGAA
cheB-r	CATTATCTTTCCTTAGCCAGCC
cobW-r	GCGCTGATATCAAGAGAGGG
cobW-f	ACTCGAAAAAACTCCTTGTCGC
AJ-r	GCGCGTGGTAATTGGAATG
AJ-f	GGTTACCGGCTTTTAAACAG

* The underlines are the site of restriction endonuclease

Table S2. List of the genes regulated by Fis

Accession no.	Gene name	Fold change	Qval	Functions
Zeamines biosynthesis				
AJC65775.1	*zmsP*	-2.94	0	HlyD family secretion protein
AJC65776.1	*zmsQ*	-2.67	0	ABC transporter ATP-binding protein
AJC65777.1	*zmsR*	-3.27	0	Hypothetical protein
AJC65778.1	*zmsA*	-4.23	0	Polyketide synthase; PKS
AJC65779.1	*zmsB*	-3.45	0	Polyunsaturated fatty acid synthase/Polyketide synthase
AJC65780.1	*zmsC*	-3.48	0	2-nitropropane dioxygenase
AJC65781.1	*zmsD*	-2.87	1.79E-77	3-oxoacyl-ACP reductase
AJC65782.1	*zmsE*	-3.68	6.38E-274	Thioester reductase
AJC65783.1	*zmsF*	-2.77	4.80E-125	Carbon-nitrogen hydrolase
AJC65787.1	*zmsJ*	-2.18	2.05E-173	Condensation protein
AJC65788.1	*zmsK*	-2.79	1.20E-167	ABC transporter ATP-binding protein
AJC65789.1	*zmsL*	-4.05	4.59E-182	ABC transporter permease
Cell wall degrading enzymes				
AJC66868.1	*celZ*	-2.49	5.98E-116	Endoglucanase
AJC66869.1	*pelL*	-2.08	1.87E-18	Pectate lyase
AJC67946.1	*pelC*	6.97	0	Pectate lyase
AJC67947.1	*pelB*	3.57	0	Pectate lyase
AJC65107.1	*pnl*	-2.67	1.61E-15	Pectate lyase
AJC66334.1	*prtC*	-2.29	6.02E-119	serralysin
AJC66335.1	*prtB*	-6.36	0	serralysin
AJC66339.1	*Inh*	-2.63	1.24E-21	Proteinase inhibitor
AJC66340.1	*prtG*	-2.89	0	Serine 3-dehydrogenase
EPS synthesis				
AJC65701.1	*wza*	13.01	0	Polysaccharide export protein Wza
AJC65702.1	*amsI*	10.29	6.39E-62	Protein-tyrosine-phosphatase
AJC65703.1	*amsA*	10.44	0	Tyrosine kinase
AJC65705.1		6.16	7.40E-108	Glycosyl transferase family 1
AJC65706.1	*rfbU*	16.13	9.62E-186	Mannosyl transferase
AJC65707.1	*rfbN*	12.27	7.02E-66	Rhamnosyltransferase
AJC65709.1	*rfbB*	2.74	4.65E-125	dTDP-glucose 4,6-dehydratase
AJC65710.1	*rfbA*	2.41	1.55E-88	Glucose-1-phosphate thymidylyltransferase
AJC65711.1	*rfbC*	2.25	8.43E-74	dTDP-4-dehydrorhamnose 3,5-epimerase
AJC65712.1	*rfbD*	2.40	6.42E-61	dTDP-4-dehydrorhamnose 3,5-epimerase
AJC65713.1	*rfbG*	4.53	5.32E-86	Glycosyl transferase family 2
AJC65715.1		9.06	3.51E-93	Glycosyl transferase
AJC65716.1	*manC*	24.25	0	Mannose-1-phosphate guanyltransferase
AJC65717.1	*manB*	2.13	2.12E-50	Phosphomannomutase
Secretion system				
T1SS				
AJC66336.1	*prtF*	-4.41	1.62E-113	Outer membrane protein
AJC66337.1	*prtE*	-2.12	1.10E-26	Protease secretion protein

Accession no.	Gene name	Fold change	Qval	Functions
T2SS				
AJC67098.1	*outK*	-2.03	1.09E-20	Type II secretion protein K
AJC67099.1	*outJ*	-3.00	8.17E-22	General secretion pathway protein J
AJC67100.1	*outI*	-3.67	7.88E-19	General secretion pathway protein I
AJC67101.1	*outH*	-2.88	2.17E-26	Type II secretion protein H
AJC67102.1	*outG*	-4.41	7.18E-184	General secretion pathway protein G
AJC67103.1	*outF*	-2.57	2.23E-43	General secretion pathway protein F
AJC67104.1	*outE*	-2.38	3.50E-86	General secretion pathway protein E
AJC67105.1	*outD*	-2.32	2.98E-113	General secretion pathway protein D
AJC67106.1	*outC*	-2.77	5.46E-72	General secretion pathway protein C
T3SS				
AJC66511.1	*hrpN*	-3.52	4.37E-12	Harpin hrpN
AJC66512.1	*hrpV*	2.18	0.0098693	Hypothetical protein
AJC66516.1	*hrpF*	-4.65	0.0013393	HPr kinase
AJC66522.1	*hrpA*	-4.86	8.27E-199	membrane protein
AJC66527.1	*hrpJ*	-2.30	0.0026069	type III secretion protein
AJC66529.1	*hrpQ*	-2.01	0.0003864	type III secretion protein
AJC66531.1	*hrpO*	-2.24	0.0641312	type III secretion protein
AJC66534.1	*hrcR*	-2.80	0.0004064	type III secretion protein
AJC66535.1	*hrcS*	2.66	0.0497029	EscS
AJC66536.1	*hrcT*	2.56	0.0008472	Type III secretion system protein
AJC66388.1		-3.46	3.21E-06	Type III effector protein
T4SS				
AJC65736.1	*vgrG*	-2.52	0	Type IV secretion protein Rhs
AJC65737.1	*pldA*	-3.52	0	Phospholipase
AJC65738.1		-3.16	0	Ankyrin
AJC65739.1		-3.90	0	Ankyrin
AJC65740.1		-3.25	0	Hypothetical protein
AJC65741.1		-2.80	2.02E-243	Hypothetical protein
AJC65742.1		-2.11	0	Hypothetical protein
AJC65745.1		-2.53	0.000591	Hypothetical protein
AJC65746.1	*rhsA*	-2.16	1.66E-07	Type IV secretion protein Rhs
AJC65747.1		-2.13	4.01E-13	Hypothetical protein
T6SS				
AJC65756.1	*impI*	-2.25	2.86E-87	Type VI secretion protein
AJC65757.1	*vasD*	-2.46	5.81E-42	Type VI secretion lipoprotein
AJC65758.1	*impJ*	-2.18	4.78E-65	Type VI secretion system protein ImpJ
Vfm quorum sensing signals				
AJC64647.1	*vfmD*	-3.63	8.57E-139	Hydrolase
AJC64648.1	*vfmE*	-14.69	0	AraC family transcriptional regulator
AJC64649.1	*vfmF*	-2.73	3.90E-72	Phosphonate ABC transporter ATP-binding protein
AJC64650.1	*vfmG*	-2.02	2.04E-80	Peptide ABC transporter permease
AJC64662.1	*vfmP*	3.26	1.31E-59	D-alanine--poly(phosphoribitol) ligase

Accession no.	Gene name	Fold change	Qval	Functions
AJC64663.1	*vfmO*	3.07	7.38E-55	D-alanine--poly(phosphoribitol) ligase
AJC64664.1	*vfmN*	5.28	5.65E-23	Hypothetical protein
AJC64665.1	*vfmM*	2.61	7.12E-38	Long-chain-fatty-acid--CoA ligase
AJC64666.1	*vfmL*	2.38	6.91E-14	Hypothetical protein
Chemotaxis protein				
AJC65328.1	*cheY*	-2.29	5.53E-34	Chemotaxis protein CheY
AJC65842.1	*cheX*	-3.18	3.42E-16	Chemotaxis protein CheX
AJC66063.1		-3.09	5.08E-169	Methyl-accepting chemotaxis protein
AJC66855.1	*cheB*	-2.04	0	Chemotaxis protein
AJC66982.1		-2.76	1.17E-139	Methyl-accepting chemotaxis protein
AJC67164.1		2.87	0	Chemotaxis protein
AJC67591.1		2.23	1.30E-220	Methyl-accepting chemotaxis protein
AJC68205.1		2.58	0	Chemotaxis protein
Tranpoter				
AJC65000.1	*mglA*	2.08	1.17E-06	Xylose ABC transporter ATP-binding protein
AJC65894.1	*phnE*	2.30	0.0039121	Phosphonate ABC transporter permease
AJC64601.1		2.08	0.0004852	Peptide ABC transporter permease
AJC64678.1		2.30	0.0001352	peptide ABC transporter substrate-binding protein
AJC64699.1	*gsiA*	2.08	0	Glutathione ABC transporter ATP-binding protein
AJC64802.1	*bztD*	2.21	9.90E-122	Arginine ABC transporter ATP-binding protein
AJC64805.1	*bztA*	2.31	5.53E-62	Amino acid ABC transporter substrate-binding protein
AJC64902.1	*exbB*	-2.74	1.72E-33	Biopolymer transporter ExbB
AJC64903.1	*exbD*	-2.18	1.15E-11	Biopolymer transporter ExbD
AJC64909.1		-2.48	3.52E-23	MFS transporter
AJC65018.1		-2.00	1.72E-15	Potassium transporter TrkG
AJC65268.1		-3.00	4.03E-05	Transporter
AJC66045.1		2.07	6.29E-14	Sugar transporter
AJC65311.1	*ugpE*	2.90	0.0168646	Sugar ABC transporter permease
AJC65334.1	*yvrC*	-6.12	0	Iron ABC transporter substrate-binding protein
AJC65809.1	*ssuB*	-2.30	2.23E-79	Nitrate ABC transporter ATP-binding protein
AJC65810.1		-2.19	3.87E-221	Sulfonate ABC transporter permease
AJC65868.1	*hycB*	2.62	0	Electron transporter
AJC65873.1		2.25	0	C4-dicarboxylate ABC transporter
AJC65905.1	*cbrB*	-2.80	0.0004064	Siderophore ABC transporter permease
AJC65936.1	*metI*	-3.21	0.0032695	Metal ABC transporter permease
AJC66266.1	*oppD*	-2.47	0.0002228	Peptide ABC transporter ATP-binding protein
AJC66307.1	*araF*	2.41	4.29E-24	Arabinose ABC transporter substrate-binding protein
AJC66426.1	*togM*	-2.56	6.02E-51	Oligogalacturonide transport system permease protein
AJC66427.1	*togN*	-2.20	2.63E-56	Oligogalacturonide transport system permease protein
AJC66428.1	*togA*	-2.21	2.22E-108	Oligogalacturonide transport system ATP-binding protein
AJC66568.1	*narK*	-5.97	0	Nitrate transporter
AJC67001.1		2.57	4.11E-192	Heme ABC transporter ATP-binding protein
AJC65999.1	*fbpA*	-2.56	1.31E-129	Iron ABC transporter substrate-binding protein
AJC66000.1	*fbpB*	-2.11	7.35E-37	Iron ABC transporter permease
AJC67079.1	*fepB*	-2.26	1.06E-54	Iron ABC transporter substrate-binding protein
AJC67081.1	*fepD*	-2.93	1.94E-25	Iron ABC transporter permease

Accession no.	Gene name	Fold change	Qval	Functions
AJC66502.1	*FeoB*	-3.79	0	Iron transporter FeoB
AJC66503.1	*feoA*	-4.44	4.54E-151	Iron transporter FeoA
AJC67280.1		-2.65	1.55E-50	Iron-siderophore ABC transporter permease
AJC67281.1	*fepC*	-2.34	9.32E-42	Iron ABC transporter ATP-binding protein
AJC67828.1	*lysE*	-3.18	7.83E-24	Multidrug transporter MatE
AJC65310.1	*malK*	2.57	1.20E-05	ABC transporter
AJC64998.1	*rbsB*	2.56	1.62E-32	ABC transporter substrate-binding protein
AJC67121.1	*livH*	2.25	0.0001321	ABC transporter permease
AJC67279.1		-3.37	1.21E-217	ABC transporter substrate-binding protein
AJC67282.1		-2.38	9.12E-17	ABC transporter substrate-binding protein
AJC67570.1		2.08	0.0012844	ABC transporter permease
AJC67793.1		2.04	0.0026421	ABC transporter substrate-binding protein
AJC68216.1	*potC*	3.75	0.0015416	ABC transporter permease
AJC67393.1	*celB*	-3.46	1.41E-05	PTS cellbiose transporter subunit IIC
AJC67395.1	*celC*	-2.08	0.0771804	PTS mannose transporter subunit IIA
AJC67561.1	*celA*	-2.33	3.70E-16	PTS cellobiose transporter subunit IIB
AJC66413.1	*manX*	2.54	0	PTS mannose transporter subunit IIAB
AJC66414.1	*manY*	2.14	5.71E-252	PTS mannose transporter subunit IIC
AJC66415.1	*manZ*	2.15	0	PTS mannose transporter subunit IID
AJC67936.1	*ulaB*	2.07	5.06E-46	PTS ascorbate transporter subunit IIBC
Membrane protein				
AJC64703.1		2.08	5.59E-20	Membrane protein
AJC65019.1		-2.60	3.50E-28	Membrane protein
AJC65094.1		2.41	1.56E-19	Membrane protein
AJC65095.1	*elaB*	2.38	2.09E-67	Membrane protein
AJC65327.1	*mipA*	-2.07	0	Membrane protein
AJC65588.1		-2.01	3.65E-25	Membrane protein
AJC66375.1		2.21	0	Membrane protein
AJC66652.1		2.25	5.13E-46	Membrane protein
AJC66796.1		-2.07	9.95E-90	Membrane protein
AJC66876.1	*flk*	-2.58	1.82E-179	Membrane protein
AJC67231.1		-2.01	2.76E-21	Membrane protein
AJC67298.1		2.14	3.37E-64	Membrane protein
AJC67589.1		2.54	3.38E-23	Membrane protein
AJC68144.1		2.10	0	Membrane protein
Tail protein				
AJC66587.1		-2.64	4.49E-08	Tail fiber protein
AJC66596.1		-2.25	8.23E-05	Tail protein
AJC66894.1		2.04	0.3951099	Tail protein
AJC66899.1		4.51	8.67E-08	Tail protein
AJC66906.1		2.68	0.0119807	Tail protein
AJC66909.1		-3.35	0.0020631	Tail protein
AJC66910.1		-3.91	0.0063559	Tail protein
AJC66911.1		-2.40	1.63E-05	Tail protein
AJC67600.1		-2.15	1.75E-16	Tail protein
AJC68118.1		-2.40	0.000543	Tail protein

Accession no.	Gene name	Fold change	Qval	Functions
Transcriptional regulator				
AJC66570.1	*narL*	-2.50	1.97E-147	Transcriptional regulator
AJC64976.1		-6.07	6.87E-06	AraC family transcriptional regulator
AJC66567.1	*acrR*	-3.22	6.87E-06	TetR family transcriptional regulator
AJC66654.1		3.02	6.31E-98	Transcriptional regulator
AJC67942.1		-2.68	3.00E-41	Transcriptional regulator
AJC68091.1		2.02	3.09E-206	Transcriptional regulator
DNA-binding protein				
AJC66259.1		-2.42	0	DNA-binding protein
AJC66879.1		-2.57	0.0163455	DNA-binding protein
AJC68179.1		2.59	7.06E-21	DNA-binding protein
Stress				
AJC64812.1	*dmsA*	2.04	0	DMSO reductase subunit A
AJC65051.1		2.35	4.15E-23	Stress-response protein
AJC65533.1		2.26	0	Heat shock protein 90
AJC66007.1		5.63	0	Heat shick protein
AJC66363.1		2.44	0	Universal stress protein E
AJC68191.1		3.15	8.20E-100	Heat-shock protein
AJC68192.1	*ibpA*	4.99	2.78E-173	Heat shock protein IbpA
Ribosomal protein				
AJC64711.1	*rpmB*	-2.17	0	50S ribosomal protein L28
AJC64774.1	*rplK*	-2.17	0	50S ribosomal protein L11
AJC64775.1	*rplA*	-2.10	0	50S ribosomal protein L1
AJC64776.1	*rplJ*	-2.03	0	50S ribosomal protein L10
AJC64865.1	*rplM*	-2.10	0	50S ribosomal protein L13
AJC65121.1	*rpsU*	-2.25	0	30S ribosomal protein S21
AJC65146.1	*rpsO*	-2.65	0	30S ribosomal protein S15
AJC65450.1	*rplS*	-2.37	0	50S ribosomal protein L19
AJC65519.1	*rpmJ*	-2.40	5.72E-10	50S ribosomal protein L36
AJC66032.1	*rplY*	-2.09	0	50S ribosomal protein L25
AJC66442.1	*rpmI*	-2.00	0	50S ribosomal protein L35
AJC67528.1	*rpsR*	-2.44	0	30S ribosomal protein S18
AJC67872.1	*rpsD*	-2.18	0	30S ribosomal protein S4
AJC67887.1	*rpsQ*	-2.45	0	30S ribosomal protein S17
AJC67888.1	*rpmC*	-2.53	1.51E-228	50S ribosomal protein L29
AJC67889.1	*rplP*	-3.17	0	50S ribosomal protein L16
AJC67890.1	*rpsC*	-3.02	0	30S ribosomal protein S3
AJC67891.1	*rplV*	-2.68	0	50S ribosomal protein L22
AJC67892.1	*rpsS*	-2.32	0	30S ribosomal protein S19
AJC67893.1	*rplB*	-2.36	0	50S ribosomal protein L2
AJC67894.1	*rplW*	-2.63	0	50S ribosomal protein L23
AJC67895.1	*rplD*	-2.46	0	50S ribosomal protein L4
AJC67896.1	*rplC*	-2.53	0	50S ribosomal protein L3
AJC67897.1	*rpsJ*	-2.09	0	30S ribosomal protein S10

Accession no.	Gene name	Fold change	Qval	Functions
AJC68307.1	*rpmH*	-2.09	0	50S ribosomal protein L34
Hypothetical protein				
AJC64584.1		2.33	5.90E-07	Hypothetical protein
AJC64701.1		4.17	0	Hypothetical protein
AJC64806.1		2.68	6.23E-159	Hypothetical protein
AJC64956.1		-2.69	0.0003123	Hypothetical protein
AJC65050.1		3.17	0	Hypothetical protein
AJC65063.1		3.76	8.76E-133	Hypothetical protein
AJC65471.1		2.07	3.05E-26	Hypothetical protein
AJC65609.1		2.23	0	Hypothetical protein
AJC65686.1		-2.74	0	Hypothetical protein
AJC65723.1		21.18	0	Hypothetical protein
AJC65724.1		2.13	8.32E-09	Hypothetical protein
AJC65727.1		2.87	2.00E-36	Hypothetical protein
AJC65733.1		-2.93	1.14E-07	Hypothetical protein
AJC65740.1		-3.25	0	Hypothetical protein
AJC65741.1		-2.80	2.02E-243	Hypothetical protein
AJC65742.1		-2.11	0	hypothetical protein
AJC65745.1		-2.53	0.000591	Hypothetical protein
AJC65747.1		-2.13	4.01E-13	Hypothetical protein
AJC65843.1		-2.41	0.0050053	Hypothetical protein
AJC65937.1		-2.83	0	Hypothetical protein
AJC65961.1		-2.64	7.28E-242	Hypothetical protein
AJC65962.1		-3.07	4.03E-115	Hypothetical protein
AJC66009.1		-9.78	0.0041324	Hypothetical protein
AJC66033.1		2.51	1.75E-265	Hypothetical protein
AJC66034.1		3.39	0	Hypothetical protein
AJC66128.1		-3.15	4.09E-95	Hypothetical protein
AJC66158.1		-2.07	4.23E-34	Hypothetical protein
AJC66175.1		-2.10	0	Hypothetical protein
AJC66182.1		-2.12	1.13E-113	Hypothetical protein
AJC66216.1		2.36	0	Hypothetical protein
AJC66223.1		2.51	0	Hypothetical protein
AJC66398.1		-2.03	1.73E-107	Hypothetical protein
AJC66420.1		2.81	0	Hypothetical protein
AJC66462.1		-3.26	0.0504403	Hypothetical protein
AJC66464.1		-23.48	0.0004777	Hypothetical protein
AJC66471.1		-2.10	2.21E-71	Hypothetical protein
AJC66490.1		-2.20	0.01717353	Hypothetical protein
AJC66501.1		-3.17	3.32E-82	Hypothetical protein
AJC66508.1		-2.05	0.00525998	Hypothetical protein
AJC66551.1		-2.58	8.48E-181	Hypothetical protein
AJC66583.1		2.28	0.010219	Hypothetical protein
AJC66653.1		3.25	1.14E-157	Hypothetical protein
AJC66700.1		2.01	4.06E-138	Hypothetical protein
AJC66708.1		-2.71	1.29E-21	Hypothetical protein
AJC66755.1		2.66	0	Hypothetical protein

Accession no.	Gene name	Fold change	Qval	Functions
AJC66777.1		-2.24	0	Hypothetical protein
AJC66880.1		-3.42	0.0092263	Hypothetical protein
AJC66882.1		-3.91	0.0172436	Hypothetical protein
AJC66885.1		-2.53	2.06E-270	Hypothetical protein
AJC66887.1		-2.18	3.63E-76	Hypothetical protein
AJC66917.1		-2.48	0	Hypothetical protein
AJC66922.1		-2.71	4.05E-117	Hypothetical protein
AJC66924.1		-3.45	0	Hypothetical protein
AJC66925.1		-3.72	0	Hypothetical protein
AJC66926.1		-3.09	0	Hypothetical protein
AJC66960.1		-2.06	2.49E-246	Hypothetical protein
AJC67176.1		2.54	0	Hypothetical protein
AJC67182.1		2.30	2.86E-55	Hypothetical protein
AJC67230.1		-5.53	4.46E-32	Hypothetical protein
AJC67234.1		-2.42	9.60E-64	Hypothetical protein
AJC67511.1		-2.00	3.47E-176	Hypothetical protein
AJC67519.1		2.59	0	Hypothetical protein
AJC67598.1		-5.66	1.13E-12	Hypothetical protein
AJC67599.1		-5.10	3.33E-14	Hypothetical protein
AJC67609.1		-2.03	1.70E-06	Hypothetical protein
AJC67696.1		-2.10	0.0009541	Hypothetical protein
AJC67697.1		-6.49	2.26E-20	Hypothetical protein
AJC67698.1		-2.12	7.80E-75	Hypothetical protein
AJC67813.1		-2.19	1.12E-47	Hypothetical protein
AJC67816.1		-5.96	9.58E-168	Hypothetical protein
AJC67943.1		-2.76	3.15E-194	Hypothetical protein
AJC68312.1		-2.53	0	Hypothetical protein
AJC68315.1		2.18	3.81E-125	Hypothetical protein
AJC68320.1		-2.33	4.30E-16	Hypothetical protein
AJC68323.1		3.39	1.87E-49	Hypothetical protein
AJC68327.1		-3.13	2.91E-05	Hypothetical protein
AJC68351.1		-2.01	3.23E-12	Hypothetical protein
AJC68373.1		-2.41	5.67E-16	Hypothetical protein
AJC68374.1		2.04	0.005476	Hypothetical protein
AJC68376.1		-4.15	0	Hypothetical protein
AJC68377.1		-8.55	1.97E-129	Hypothetical protein
AJC68380.1		-2.02	4.45E-05	Hypothetical protein
AJC68389.1	*rhtB*	-5.33	5.95E-08	Hypothetical protein
AJC68391.1		-3.49	2.51E-77	Hypothetical protein
AJC68394.1		-2.05	1.46E-05	Hypothetical protein
AJC68397.1		-3.48	2.81E-07	Hypothetical protein
AJC68406.1		2.14	1.45E-08	Hypothetical protein
AJC68411.1		3.17	0.0006215	Hypothetical protein
AJC68422.1		-3.42	0.0172686	Hypothetical protein
AJC68427.1		2.30	0.1491387	Hypothetical protein
AJC68434.1		4.08	0	Hypothetical protein
AJC68442.1		-3.22	3.55E-206	Hypothetical protein
AJC68452.1		-4.18	1.27E-13	Hypothetical protein

Accession no.	Gene name	Fold change	Qval	Functions
AJC68470.1		-2.45	0.2607386	Hypothetical protein
AJC68474.1		-2.08	0.0004838	Hypothetical protein
AJC68479.1		-3.21	1.85E-98	Hypothetical protein
Others				
AJC65065.1		2.10	1.05E-20	OHCU decarboxylase
AJC65896.1	*ectB*	-3.49	1.43E-32	Diadenosine tetraphosphatase
AJC65897.1	*cirA*	-4.61	8.51E-113	TonB-denpendent receptor
ACT08493.1	*insA*	-2.89	6.70E-135	Insertion element protein
AJC64583.1		2.10	0	Oligopeptidase A
AJC64609.1		2.04	2.30E-29	DNA-3-methyladenine glycosylase
AJC64617.1		4.09	1.90E-12	lauroyl acyltransferase
AJC64642.1	*indC*	-2.05	2.71E-142	Indigoidine synthase
AJC64691.1	*tolC*	-2.77	5.64E-35	Outer membrane protein TolC
AJC64700.1		3.13	6.63E-301	Dipeptide epimerase
AJC64704.1		3.65	0	Ribonuclease PH
AJC64705.1	*pyrE*	2.65	8.81E-43	Orotate phosphoribosyltransferase
AJC64738.1		-2.78	1.35E-112	5-methyltetrahydropteroyltriglutamate --homocysteine methyltransferase
AJC64764.1		2.00	3.55E-253	Proline dipeptidase
AJC64769.1	*birA*	-2.03	5.05E-21	Bifunctional biotin--[acetyl-CoA-carboxylase] Synthetase/biotin
AJC64771.1	*tuf*	-4.44	0	Elongation factor Tu
AJC64779.1	*rpoC*	-2.00	0	DNA-directed RNA polymerase subunit beta
AJC64782.1	*yedY*	2.04	5.98E-10	Molybdopterin binding oxidoreductase
AJC64783.1		4.55	2.57E-59	Thioredoxin reductase
AJC64784.1		2.09	1.59E-07	Hypothetical protein
AJC64787.1	*thiH*	-2.31	8.77E-41	Thiamine biosynthesis protein ThiH
AJC64789.1	*thiS*	-3.91	3.30E-07	Sulfur carrier protein ThiS
AJC64813.1		2.51	2.90E-142	Dehydrogenase
AJC64825.1	*yhdh*	2.33	0	Quinone oxidoreductase
AJC64835.1		-2.93	0.0026469	Electron transfer flavoprotein FixA
AJC64907.1		2.70	3.93E-122	Sucrose porin
AJC64908.1		2.28	1.55E-30	Aminoimidazole riboside kinase
AJC64930.1		-2.41	6.05E-07	Calcium sensor EFh
AJC64940.1		2.61	5.19E-74	Ornithine carbamoyltransferase
AJC64953.1		-2.34	0	NgoFVII restriction endonuclease domain protein
AJC64966.1		-2.26	0	Dynamin family protein
AJC64968.1	*rssA*	-2.20	4.69E-118	Phospholipase
AJC65002.1		2.87	2.49E-19	Carbohydrate kinase
AJC65052.1	*tatD*	2.34	7.27E-44	Deoxyribonuclease
AJC65081.1	*groES*	2.30	6.13E-127	Co-chaperonin GroES
AJC65082.1	*groEL*	2.76	0	Molecular chaperone GroEL
AJC65112.1	*uhpT*	-2.48	3.76E-84	Antiporter
AJC65114.1		2.07	8.61E-83	Thiamine biosynthesis lipoprotein ApbE
AJC65118.1		2.39	2.37E-13	GNAT family acetyltraansferase
AJC65124.1		2.00	0	Malate dehydrogenase
AJC65140.1	*secG*	-2.17	0	Preprotein translocase subunit SecG

Accession no.	Gene name	Fold change	Qval	Functions
AJC65152.1		2.09	1.95E-214	Putative protease
AJC65153.1		2.20	0	Putative protease
AJC65174.1		2.26	1.92E-67	Hemolysin
AJC65217.1		-2.00	2.79E-131	Global regulatory protein
AJC65245.1	*iolE*	2.12	1.51E-24	Xylose isomerase
AJC65353.1		-6.36	0	Aldehyde dehydrogenase
AJC65391.1	*fabZ*	-2.04	3.02E-105	Hydroxymyristoyl-ACP dehydratase
AJC65448.1	*rimM*	-2.09	0	16S rRNA-processing protein M
AJC65449.1	*trmD*	-2.29	0	tRNA (guanine-N1)-methyltransferase
AJC65455.1	*raiA*	2.67	0	Cold-shock protein
AJC65515.1		3.99	0	Glycoprotein/polysaccharide metabolism
AJC65545.1		2.15	5.01E-09	2Fe-2S ferredoxin
AJC65547.1		2.36	0	Ribonucleotide-diphosphate reductase subunit alpha
AJC65592.1		-2.29	0	RNA chaperone/anti-terminator
AJC65621.1	*asnB*	2.68	0	Asparagine synthetase B
AJC65749.1		-2.39	9.47E-18	Plasmid stabilization protein
AJC65822.1		-4.78	1.53E-135	Flavin reductase
AJC65844.1		-2.05	0.0061249	Cupin
AJC65850.1		3.95	0	Aspartate racemase
AJC65858.1	*hycI*	2.03	1.63E-56	Hydrogenase 3 maturation protease
AJC65861.1	*hycF*	2.22	2.47E-87	Formate hydrogenlyase complex iron-sulfur subunit
AJC65862.1	*hycE*	2.33	0	Hydrogenase 3 large subunit
AJC65863.1	*hyfF*	2.67	1.84E-268	NADH dehydrogenase
AJC65864.1	*hyfE*	3.18	6.28E-170	Hydrogenase-4 subunit E
AJC65865.1		3.31	0	Oxidoreductase
AJC65866.1	*hycD*	2.83	2.49E-151	Hydrogenase 3 membrane subunit
AJC65867.1		2.77	0	Hydantoin racemase
AJC65882.1		2.17	0.0610483	PhnG
AJC65883.1	*phnH*	3.07	0.2970395	Phosphonate C-P lyase system protein PhnH
AJC65884.1	*PhnI*	2.04	0.0572334	Carbon-phosphorus lyase complex subunit PhnI
AJC65886.1	*phnK*	4.34	0.0028578	Phosphonate C-P lyase
AJC65890.1		2.60	0.004616	Transposase
AJC65953.1		3.39	3.26E-06	Endoribonuclease
AJC65963.1		-2.46	4.10E-06	Antitoxin
AJC66023.1		-2.53	2.69E-19	ATPase AAA
AJC66068.1	*rimO*	2.34	5.26E-135	Ribosomal protein S12 methylthiotransferase
AJC66071.1		-2.53	1.18E-06	3-hydroxydecanoyl-ACP:CoA transacylase
AJC66094.1		10.55	1.18E-06	Oxidoreductase
AJC66095.1	*hcp*	14.10	0	Hydroxylamine reductase
AJC66142.1	*mhpD*	2.05	4.48E-34	5-oxopent-3-ene-1,2,5-tricarboxylate decarboxylase
AJC66143.1		709.38	0.0014184	[citrate (pro-3S)-lyase] ligase
AJC66181.1		-2.20	1.12E-157	Alanine acetyltransferase
AJC66189.1		-2.02	0.0207901	Glycerol acyltransferase
AJC66221.1	*purT*	-2.04	2.14E-43	Phosphoribosylglycinamide formyltransferase
AJC66239.1		2.27	0	PrkA family serine protein kinase
AJC66261.1		2.81	0	Acetaldehyde dehydrogenase
AJC66273.1	*tonB*	-2.48	3.23E-57	Cell envelope protein TonB
AJC66305.1	*araA*	3.99	3.35E-77	Arabinose isomerase

Accession no.	Gene name	Fold change	Qval	Functions
AJC66306.1	*araB*	3.39	1.32E-34	Ribulokinase
AJC66327.1		2.05	8.30E-30	Glycosyl transferase
AJC66393.1		2.09	0	SpoVR family protein
AJC66410.1		2.45	1.59E-175	NUDIX hydrolase
AJC66463.1		-19.57	8.71E-06	Host-nuclease inhibitor protein Gam
AJC66481.1		2.73	0.0001209	Phage morphogeneis protein
AJC66491.1		2.16	1.32E-05	Phage tail tape measure protein
AJC66552.1		-2.50	6.05E-98	5-methyltetrahydropteroyltriglutamate --homocysteine methyltransferase
AJC66562.1	*narI*	-5.31	0	Nitrate reductase A subunit gamma
AJC66563.1	*narJ*	-6.85	0	Nitrate reductase molybdenum cofactor Assembly chaperone NarJ
AJC66564.1	*narH*	-7.00	0	Nitrate reductase A subunit beta
AJC66565.1	*narZ*	-9.85	0	Nitrate reductase A subunit alpha
AJC66569.1	*narX*	-3.84	0	Nitrate/nitrite sensor protein NarX
AJC66605.1		-2.29	3.50E-18	Late control D family protein
AJC66655.1	*pspB*	2.87	1.75E-61	Phage-shock protein
AJC66656.1		4.09	0	Phage-shock protein
AJC66673.1	*araD*	2.59	5.64E-14	L-ribulose-5-phosphate 4-epimerase
AJC66680.1	*ribA*	-2.09	8.28E-234	GTP cyclohydrolase II
AJC66705.1		2.30	0.0413387	Transposase IS4
AJC66771.1	*acpP*	-2.52	0	Acyl carrier protein
AJC66772.1	*fabG*	-2.26	0	3-ketoacyl-ACP reductase
AJC66883.1		-5.38	2.74E-10	DNA adenine methylase
AJC66896.1		2.56	0.0952266	Lysozyme
AJC66923.1		-2.60	0	PAAR motif family protein
AJC66927.1	*rhs*	-2.60	0	Type IV secretion protein Rhs
AJC66939.1	*virB2*	2.04	0.3951099	Conjugal transfer protein
AJC66950.1		-2.46	2.99E-80	Molybdopterin-guanine dinucleotide biosynthesis protein MobB
AJC67029.1	*yfcH*	2.37	0	Epimerase
AJC67074.1		-2.05	2.10E-186	Long-chain fatty acid outer membrane transporter
AJC67112.1		-2.17	1.03E-07	Transposase IS3
AJC67127.1		-2.03	0.000122	Histidine kinase
AJC67196.1		-2.22	8.28E-14	Protein sxy
AJC67226.1		-4.98	1.05E-156	Short-chain dehydrogenase
AJC67227.1		-5.20	6.36E-176	FAD-dependent oxidoreductase
AJC67229.1	*cfa*	-4.91	7.13E-118	Cyclopropane-fatty-acyl-phospholipid synthase
AJC67304.1		-2.18	2.57E-171	Diguanylate cyclase
AJC67361.1		-2.38	4.56E-288	5-methyltetrahydropteroyltriglutamate --homocysteine methyltransferase
AJC67372.1		2.01	0	L-asparaginase
AJC67391.1		-2.32	2.99E-48	Beta-D-glucuronidase
AJC67392.1	*oprD*	-8.53	5.63E-12	Porin
AJC67397.1	*purE*	3.43	0	2-dehydropantoate 2-reductase
AJC67448.1		2.12	0	Protein lysine acetyltransferase
AJC67489.1		2.08	5.97E-111	Alpha-dehydro-beta-deoxy-D-glucarate aldolase
AJC67490.1	*gudD*	2.31	1.09E-200	Glucarate dehydratase

Accession no.	Gene name	Fold change	Qval	Functions
AJC67491.1	*gudD*	2.36	2.44E-124	Glucarate dehydratase
AJC67493.1	*garD*	2.22	0	Galactarate dehydrogenase
AJC67495.1		2.45	0	FlxA protein
AJC67518.1		2.08	8.17E-45	Iron-sulfur cluster repair di-iron protein
AJC67529.1		-2.11	0	Primosomal replication protein N
AJC67542.1	*pilT*	2.98	2.74E-32	Pilus retraction ATPase PilT
AJC67579.1		-2.86	1.64E-278	DEAD/DEAH box helicase
AJC67581.1		5.37	3.09E-258	Phage-shock protein
AJC67582.1		2.01	9.39E-254	Quinone oxidoreductase
AJC67601.1		-3.20	1.81E-14	Phage tail protein
AJC67602.1		-3.40	1.15E-06	Phage tail protein
AJC67605.1		-4.65	0.0013393	Type VI secretion protein
AJC67629.1		2.32	1.21E-06	Dihydrodipicolinate synthetase
AJC67651.1		2.76	1.23E-74	Guanosine 5'-monophosphate oxidoreductase
AJC67665.1		-2.03	4.80E-42	Ell division protein FtsW
AJC67679.1		-2.12	2.07E-193	2-isopropylmalate synthase
AJC67683.1	*viuB*	-4.45	8.66E-75	Iron utilization protein
AJC67726.1	*dnaJ*	2.13	3.98E-182	Molecular chaperone DnaJ
AJC67727.1	*dnaK*	3.35	0	Molecular chaperone DnaK
AJC67743.1	*csdA*	-2.52	2.45E-29	Aminotransferase class V
AJC67799.1		2.31	6.81E-35	Organic hydroperoxide resistance protein
AJC67817.1	*jmjC*	-4.72	1.14E-184	Hydroxylase
AJC67827.1	*paaG*	-2.20	4.42E-37	Polyketide biosynthesis enoyl-CoA hydratase
AJC67852.1	*aceK*	2.02	2.84E-18	Bifunctional isocitrate dehydrogenase kinase/phosphatase
AJC67854.1	*aceB*	2.56	8.25E-152	Malate synthase
AJC67876.1	*secY*	-2.23	0	Preprotein translocase subunit SecY
AJC67898.1		-2.17	9.28E-109	Bacterioferritin
AJC67934.1	*araD*	2.71	1.22E-17	Ribulose-5-phosphate 4-epimerase
AJC67937.1		2.32	1.48E-25	L-ascorbate 6-phosphate lactonase
AJC67944.1		-6.55	0	Peptidase S8
AJC67945.1		-2.94	4.40E-29	Glycoside hydrolase family 19
AJC67967.1		2.04	4.82E-181	Nucleotidase
AJC68007.1	*glpC*	6.54	0	sn-glycerol-3-phosphate dehydrogenase subunit C
AJC68008.1	*glpB*	3.92	0	Glycerol-3-phosphate dehydrogenase subunit B
AJC68009.1	*glpA*	3.79	0	sn-glycerol-3-phosphate dehydrogenase subunit A
AJC68011.1	*glpQ*	2.78	0	Glycerophosphodiester phosphodiesterase
AJC68030.1	*hemC*	2.03	3.05E-58	Porphobilinogen deaminase
AJC68038.1		-3.06	0.0029613	N-acetyltransferase
AJC68087.1		2.01	2.42E-73	Cystathionine gamma-synthase
AJC68093.1		2.27	7.77E-63	ATP-dependent protease
AJC68094.1	*hslU*	2.16	1.46E-202	ATP-dependent protease
AJC68240.1	*glnA*	2.86	0	Glutamine synthetase
AJC68241.1	*glnL*	2.17	9.03E-154	Nitrogen regulation protein NR(II)
AJC68296.1		-2.48	5.98E-215	UDP phosphate-alpha-4-amino-4-deoxy-L-arabinose Arabinosyl transferase
AJC68444.1		-7.83	0.0005393	Transposase IS116
AJC68483.1		-11.59	4.69E-31	PP-binding

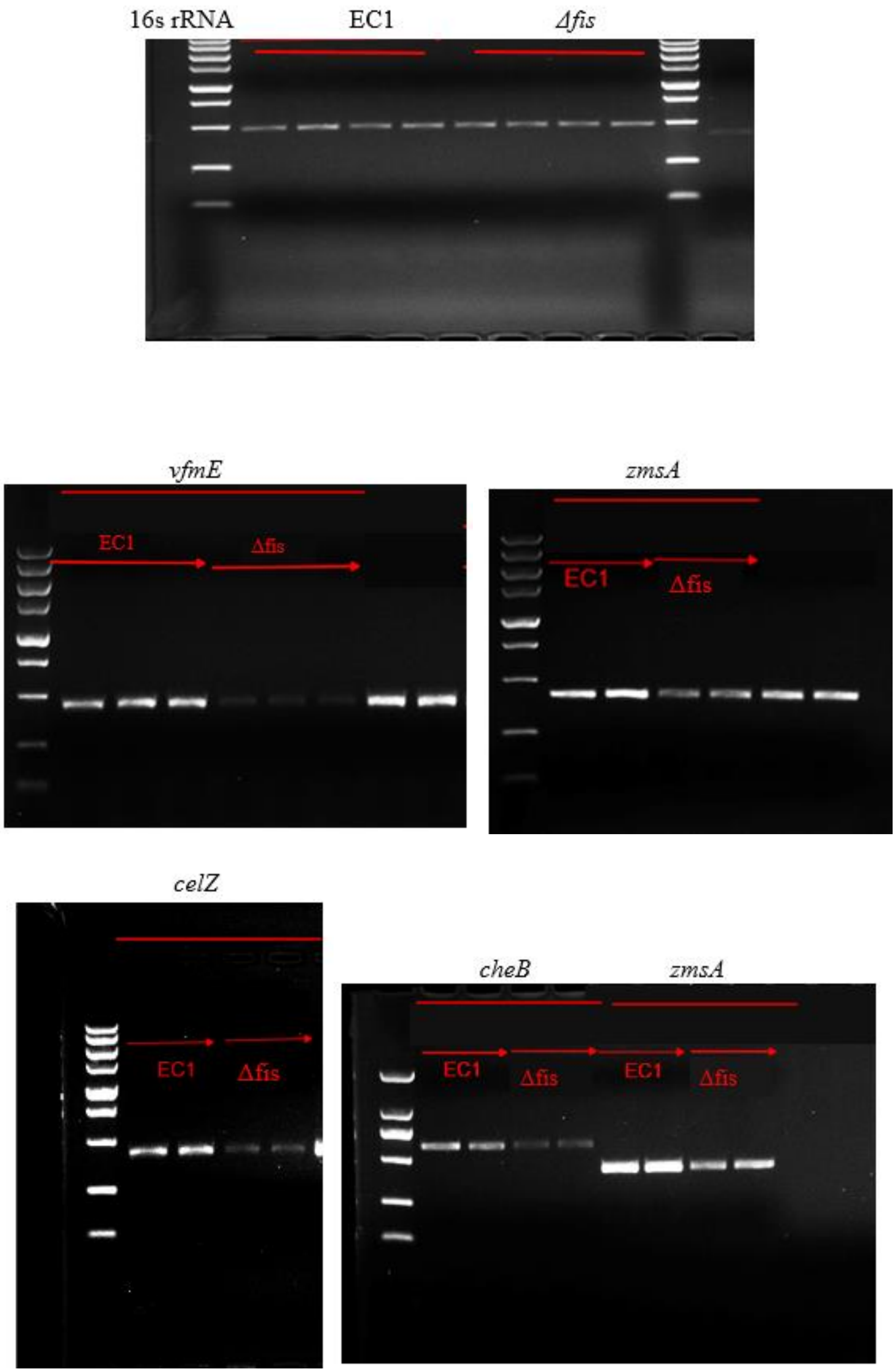
16s rRNA
EC1
Δfis
vfmE
EC1
Δfis
zmsA
EC1
Δfis
celZ
EC1
Δfis
cheB
zmsA
EC1
Δfis
EC1
Δfis

prtF

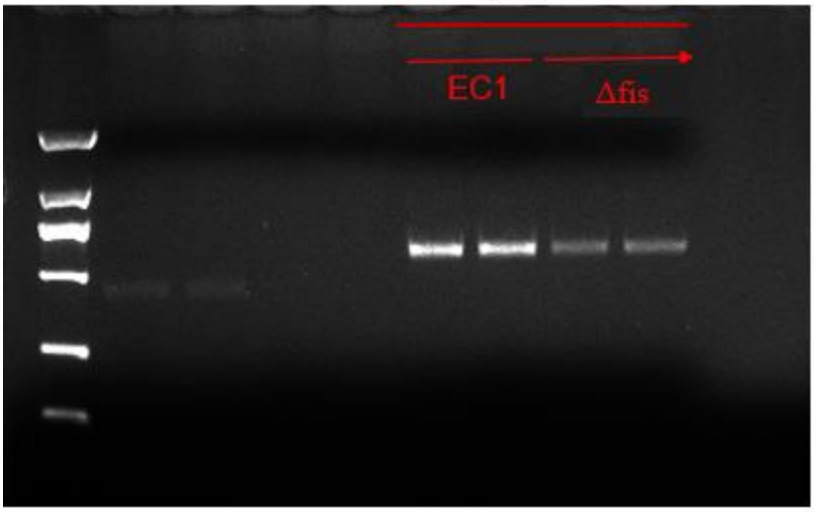

rfbU

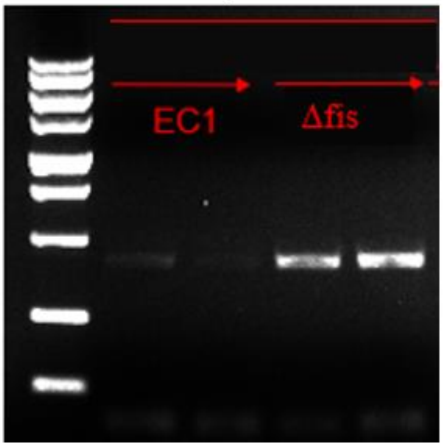

amsA

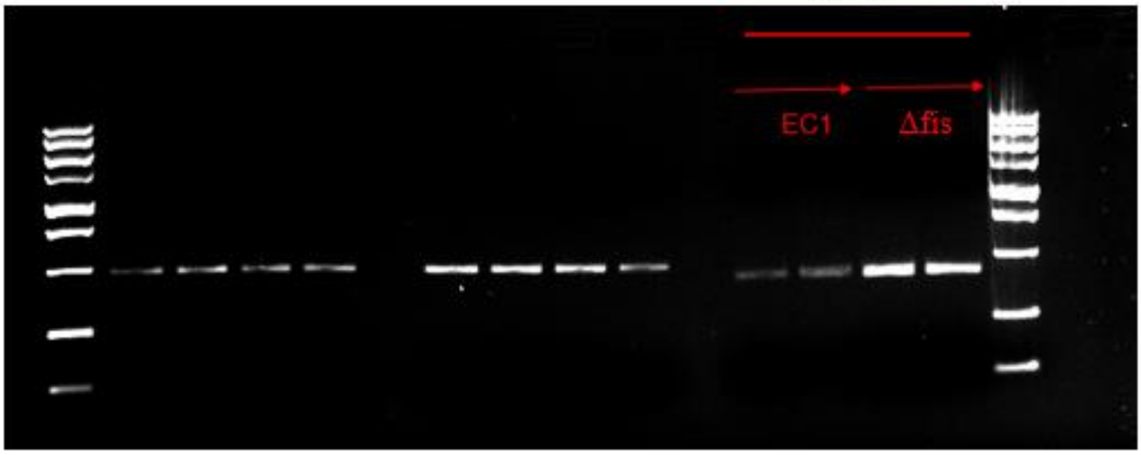

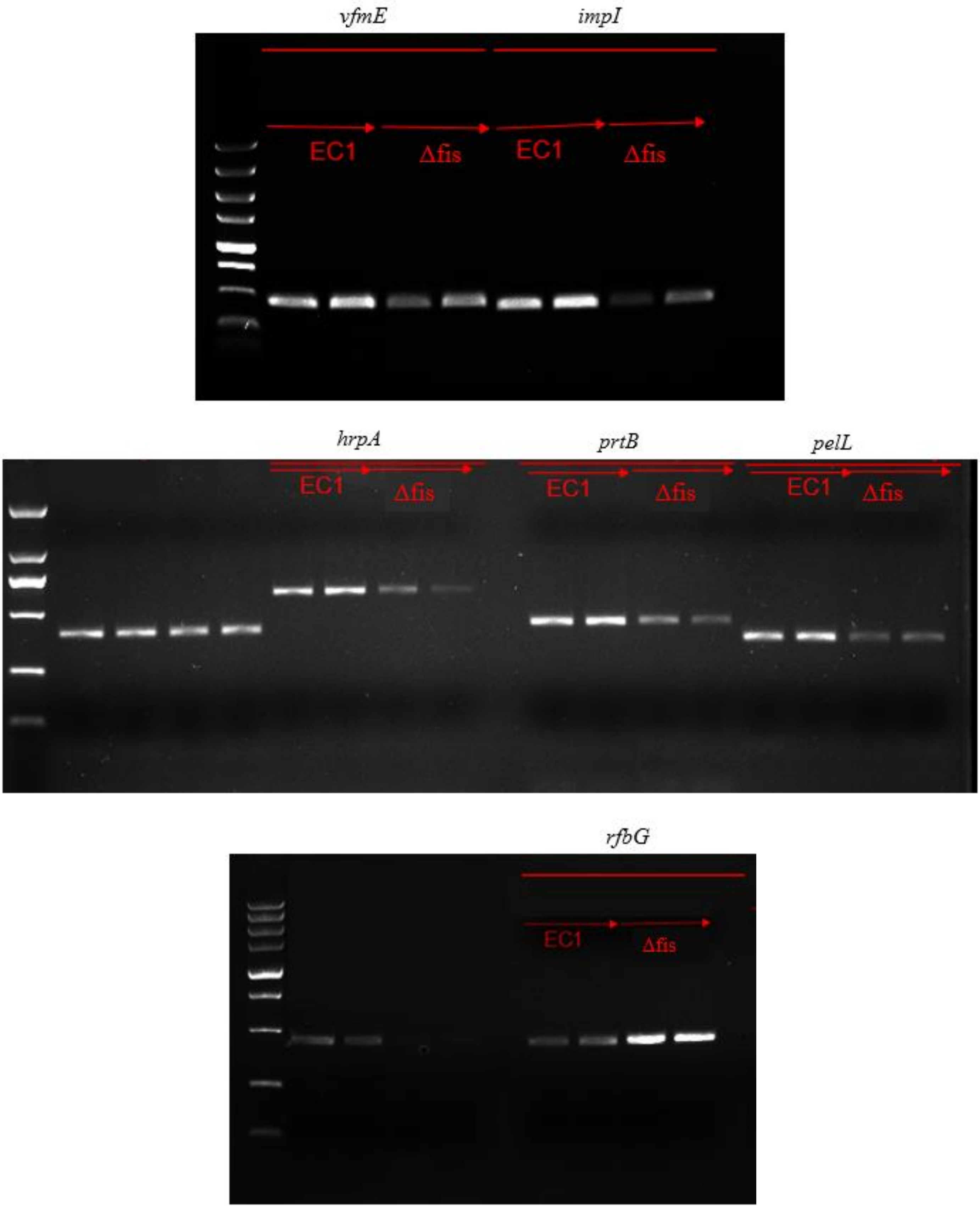

Figure S1. RT-PCR analysis of Fis on modulating the genes expression of *vfmE*, *zmsA*, *celZ*, *cheB*, *rfbU*, *amsA*, *impI*, *hrpA*, *prtB* and *pelL*. This figure shows the original photos of Figure 2.

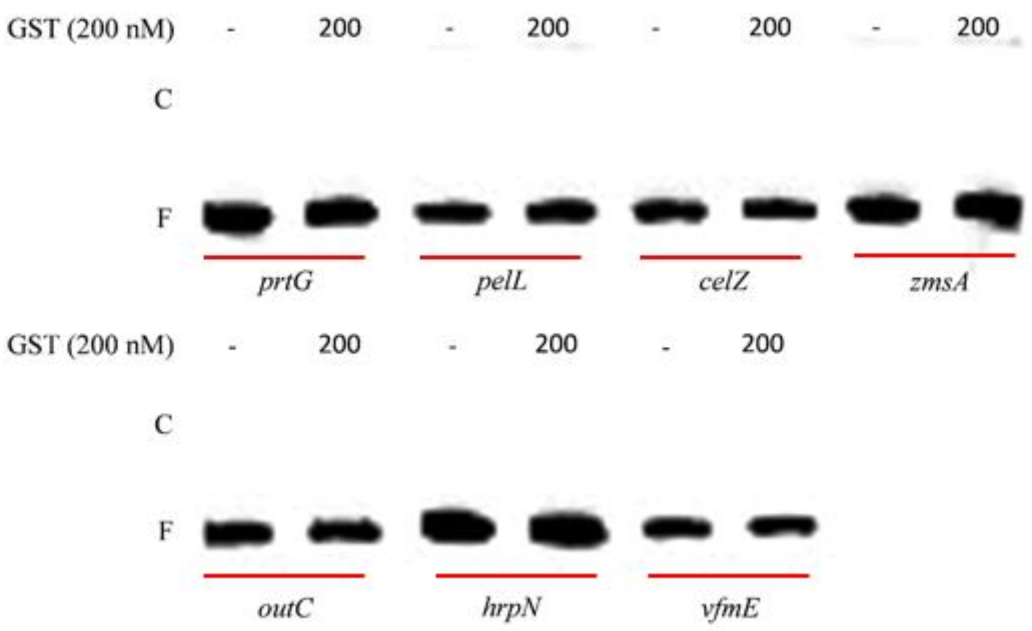

Figure S2. Binding of GST at the *prtG*, *pelL*, *celZ*, *zmsA*, *outC*, *hrpN* and *vfmE* promoters. This figure shows the original photos of Figure 8. Thirty fmol of labelled DNA sequences corresponding to promoter region of *prtG*, *pelL*, *celZ*, *zmsA*, *outC*, *hrpN* and *vfmE* genes were incubation with 200 nM GST protein, and who could not bind to the promoter region of all the genes. The position of free DNA (F) and of protein-DNA complexes (C) are shown.

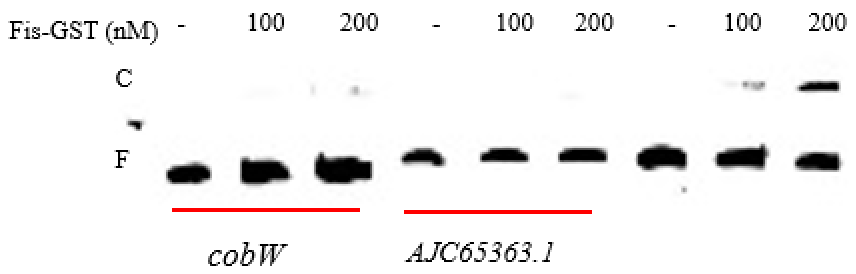
Fis-GST (nM)
-
100
200
-
100
200
-
100
200
C
F
cobW
AJC65363.1

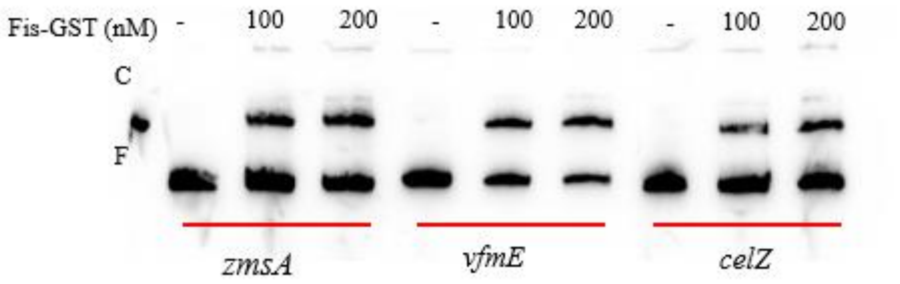
Fis-GST (nM)
-
100
200
-
100
200
-
100
200
C
F
zmsA
vfmE
celZ

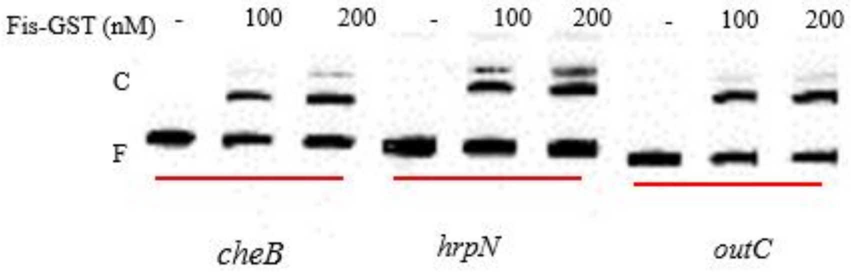
Fis-GST (nM)
-
100
200
-
100
200
-
100
200
C
F
cheB
hrpN
outC

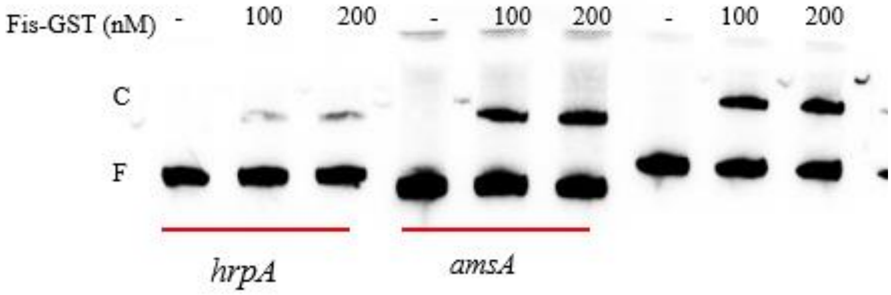
Fis-GST (nM)
-
100
200
-
100
200
-
100
200
C
F
hrpA
amsA

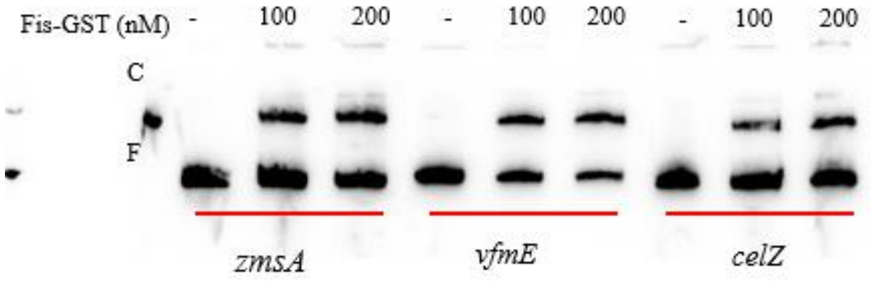
Fis-GST (nM)
-
100
200
-
100
200
-
100
200
C
F
zmsA
vfmE
celZ

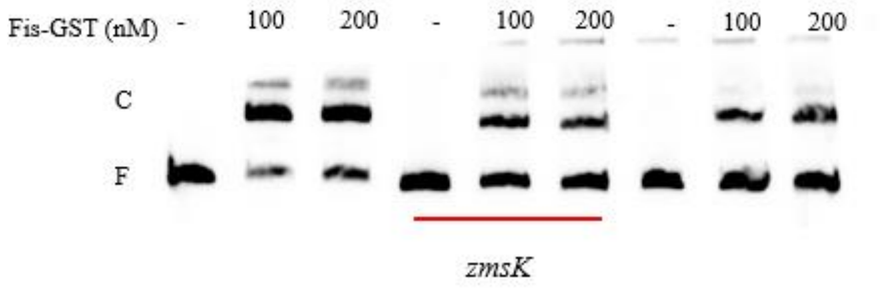

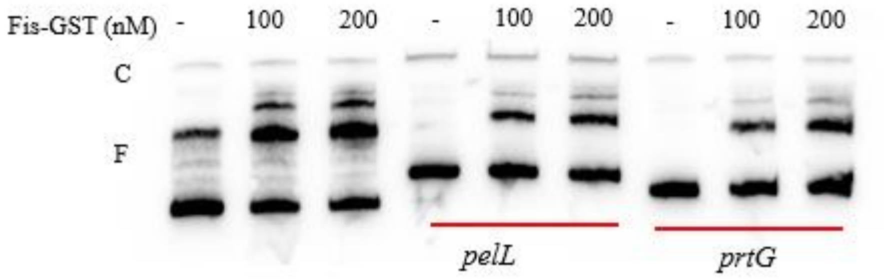

Figure S3. EMSA analysis of Fis binding at the promoter regions of *zmsA*, *zmsK*, *celZ*, *pelL*, *prtG*, *vfmE*, *hrpN*, *outC*, *amsA* and *cheB*. This figure shows the original photo of Figure 8.
